# Author Correction: Enhancing operational stability of OLEDs based on subatomic modified thermally activated delayed fluorescence compounds

**DOI:** 10.1038/s41467-023-42698-1

**Published:** 2023-10-26

**Authors:** Sinyeong Jung, Wai-Lung Cheung, Si-jie Li, Min Wang, Wansi Li, Cangyu Wang, Xiaoge Song, Guodan Wei, Qinghua Song, Season Si Chen, Wanqing Cai, Maggie Ng, Wai Kit Tang, Man-Chung Tang

**Affiliations:** 1 Institute of Materials Research, Tsinghua Shenzhen International Graduate School, Tsinghua University, 518055 Shenzhen, China; 2 Tsinghua-Berkeley Shenzhen Institute (TBSI), Tsinghua University, 518055 Shenzhen, China; 3 Institute of Environment and Ecology, Tsinghua Shenzhen International Graduate School, Tsinghua University, 518005 Shenzhen, China; 4 Faculty of Materials Science, MSU-BIT University, 518172 Shenzhen, China; 5 Department of Chemistry, Faculty of Science, University of Malaya, 50603 Kuala Lumpur, Malaysia

**Keywords:** Electronic devices, Organic LEDs

Correction to: *Nature Communications* 10.1038/s41467-023-42019-6, published online 14 October 2023

The original version of this Article contained an error in the Results section in the main text, which incorrectly read ‘This observation imparts a scholarly character to the study.’ The correct version removes this sentence. This has been corrected in both the PDF and HTML versions of the Article.

