# Supplementary material for: Enhancing operational stability of OLEDs based on subatomic modified thermally activated delayed fluorescence compounds
Source: Nat Commun. 2023 Oct 14;14:6481. doi: 10.1038/s41467-023-42019-6 (PMC10576749; doi:10.1038/s41467-023-42019-6)
Supplement: Supplementary file 1 — Supplementary Information [file 41467_2023_42019_MOESM1_ESM.pdf]

Supplementary Information

Enhancing Operational Stability of OLEDs Based on Subatomic Modified Thermally Activated Delayed Fluorescence Compounds

Sinyeong Jung,[1,2,‡] Wai-Lung Cheung,[1,‡] Si-jie Li,[1,‡] Min Wang,[1] Wansi Li,[1] Cangyu Wang,[1] Xiaoge Song,[1] Guodan Wei,[1,2,] Qinghua Song,[1] Season Si Chen,[3,*] Wanqing Cai,[4] Maggie Ng,[1] Wai Kit Tang,[5] Man-Chung Tang[1,*]*

[1] Institute of Materials Research, Tsinghua Shenzhen International Graduate School, Tsinghua University, Shenzhen 518055, China

[2] Tsinghua-Berkeley Shenzhen Institute (TBSI), Tsinghua University, Shenzhen 518055, China

[3] Institute of Environment and Ecology, Tsinghua Shenzhen International Graduate School, Tsinghua University, Shenzhen 518005, China

[4] Faculty of Materials Science, MSU-BIT University, Shenzhen 518172, China

[5] Department of Chemistry, Faculty of Science, University of Malaya, 50603 Kuala Lumpur, Malaysia

[‡] These authors contributed equally.

* E-mail: kobetang2021@sz.tsinghua.edu.cn;
season.chen@sz.tsinghua.edu.cn;
weiguodan@sz.tsinghua.edu.cn

Table of Content

Table of Content		Page
**Supplementary Fig. 1**	Synthetic route for the BO and d-BO precursor ligands.	5
**Supplementary Fig. 2**	Synthetic route for the BO and d-BO units.	6
**Supplementary Fig. 3**	Synthetic route for d-DPA.	7
**Supplementary Fig. 4**	Synthetic route for the TADF compounds.	8−9
**Supplementary Fig. 5**	^{1}H NMR spectrum of 2,5-dibromo-1,3-di(phenyloxy)benzene in $CDCl_3$.	10
**Supplementary Fig. 6**	^{1}H NMR spectrum of 2,5-dibromo-1,3-di(phenyloxy)benzene-d_5 in $CDCl_3$.	10
**Supplementary Fig. 7**	^{1}H NMR spectrum of BO in $CDCl_3$.	11
**Supplementary Fig. 8**	^{1}H NMR spectrum of d-BO in $CDCl_3$.	11
**Supplementary Fig. 9**	^{1}H NMR spectrum of d-DPA in $CDCl_3$.	12
**Supplementary Fig. 10**	^{1}H NMR spectrum of **DPA-BO** in DMSO-d_6.	12
**Supplementary Fig. 11**	^{1}H NMR spectrum of **d-DPA-BO** in DMSO-d_6.	13
**Supplementary Fig. 12**	^{1}H NMR spectrum of **DPA-d-BO** in DMSO-d_6.	13
**Supplementary Fig. 13**	^{1}H NMR spectrum of **d-DPA-d-BO** in DMSO-d_6.	14
**Supplementary Fig. 14**	^{13}C NMR spectrum of **DPA-BO** in $CDCl_3$.	14
**Supplementary Fig. 15**	^{13}C NMR spectrum of **d-DPA-BO** in $CDCl_3$.	15
**Supplementary Fig. 16**	^{13}C NMR spectrum of **DPA-d-BO** in $CDCl_3$.	15
**Supplementary Fig. 17**	^{13}C NMR spectrum of **d-DPA-d-BO** in $CDCl_3$.	16
**Supplementary Fig. 18**	High-resolution ESI-MS of **DPA-BO**.	16
**Supplementary Fig. 19**	High-resolution ESI-MS of **d-DPA-BO**.	17
**Supplementary Fig. 20**	High-resolution ESI-MS of **DPA-d-BO**.	17
**Supplementary Fig. 21**	High-resolution ESI-MS of **d-DPA-d-BO**.	18
**Supplementary Fig. 22**	IR spectra of the TADF compounds.	18
**Supplementary Fig. 23**	Thermogravimetric analysis and differential scanning calorimetry traces.	19
**Supplementary Table 1**	Thermal properties of the TADF compounds.	19
**Supplementary Fig. 24**	Spatial plots (isovalue = 0.03) of selected molecular orbitals of **DPA-BO** at the optimized ground-state geometry.	20
**Supplementary Fig. 25**	Spatial plots (isovalue = 0.03) of selected molecular orbitals of **d-DPA-BO** at the optimized ground-state geometry.	20
**Supplementary Fig. 26**	Spatial plots (isovalue = 0.03) of selected molecular orbitals of **DPA-d-BO** at the optimized ground-state geometry.	21
**Supplementary Fig. 27**	Spatial plots (isovalue = 0.03) of selected molecular orbitals of **d-DPA-d-BO** at the optimized ground-state geometry.	21
**Supplementary Fig. 28**	Simulated absorption spectra of the TADF compounds.	22
**Supplementary Table 2**	First fifteen singlet excited states (S_n) of the TADF compounds computed by TDDFT/PCM at the PBE0 level using toluene as the solvent.	23−25
**Supplementary Table 3**	Relative energies of the lowest-lying singlet excited states (S_1) of the TADF compounds optimized at the PBE0 level.	25
**Supplementary Fig. 29**	Spatial plots (isovalue = 0.03) of selected molecular orbitals of **DPA-BO** at the optimized S_1 state geometry.	26
**Supplementary Fig. 30**	Spatial plots (isovalue = 0.03) of selected molecular orbitals of **DPA-BO** at the optimized T_1 state geometry.	26

**Supplementary Fig. 31**	Spatial plots (isovalue = 0.03) of selected molecular orbitals of **DPA-BO** at the optimized T_2 state geometry.	26
**Supplementary Table 4**	Zero point energy of the compounds at the ground-state geometry optimized at the PBE0/6-31G(d,p) level.	27
**Supplementary Table 5**	Bond dissociation energy of the C−N bonds of the compounds optimized at the M06-2X/6-311++G(d,p) level.	27
**Supplementary Table 6**	Bond dissociation energy of the C−H or C−D bonds of the compounds optimized at the M06-2X/6-311++G(d,p) level.	27
**Supplementary Table 7**	Enthalpies of activation corresponding to the transition state of the C–N bond dissociation of **DPA-BO** and **d-DPA-d-BO** on the triplet potential energy surface.	27
**Supplementary Fig. 32**	Plots of computed reorganization energies as a function of normal mode wavenumbers for S_1-S_0.	28
**Supplementary Fig. 33**	Illustration of selected normal modes contributing to large reorganization energies for the S_0 state.	28
**Supplementary Fig. 34**	Illustration of selected normal modes contributing to large reorganization energies for the S_1 state.	29
**Supplementary Table 8**	Vibrational frequencies (ω_j), displacement (ΔQ), Huang-Rhys factors (S_j) and reorganization energies (λ_j) of selected normal modes for S_0 of the compounds in toluene.	29
**Supplementary Table 9**	Vibrational frequencies (ω_j), displacement (ΔQ), Huang-Rhys factors (S_j) and reorganization energies (λ_j) of selected normal modes for S_1 of the compounds in toluene.	30
**Supplementary Table 10**	The computed radiative (k_r) and nonradiative (k_{nr}) decay rate constants of the S_1 state.	30
**Supplementary Fig. 35**	Normalized PL spectra of the TADF compounds in various solvents.	31
**Supplementary Table 11**	Photophysical data of the TADF compounds.	32−33
**Supplementary Fig. 36**	Normalized PL spectra of 5−20 wt% **DPA-BO** doped in DPEPO films at 298 K.	34
**Supplementary Fig. 37**	Spatial localization of 10 wt% compounds in DPEPO.	34
**Supplementary Fig. 38**	Normalized PL spectra of 10 wt% TADF compounds doped in DPEPO films at 300 K and 77 K.	35
**Supplementary Fig. 39**	Arrhenius plots of k_{RISC} of the TADF compounds.	35
**Supplementary Fig. 40**	UPS spectra of the TADF compounds.	36
**Supplementary Fig. 41**	UV-Vis spectra of the TADF compounds in toluene solution.	37
**Supplementary Fig. 42**	PL spectra of the TADF compounds in toluene solution.	37
**Supplementary Fig. 43**	PL spectra of the TADF compounds doped in DPEPO films.	38
**Supplementary Fig. 44**	Time-dependent EPR spectra of the TADF compounds.	38
**Supplementary Fig. 45**	Device characteristics of 5−20 wt% of the compounds doped in DPEPO.	39
**Supplementary Fig. 46**	PL spectra and delayed PL decay of BN3 and BN3 + DPA-BO doped in mCBP.	40
**Supplementary Note 1**		41−42
**Supplementary Fig. 47**	Spatial localization of 3 wt% **DPA-BO** doped in BH.	43
**Supplementary Fig. 48**	Device characteristics and transient EL decay of vacuum-deposited	44

	OLEDs based on the compounds doped in BH.	
**Supplementary Fig. 49**	Device characteristics of 3 wt% of the compounds doped in BH.	45
**Supplementary Table 12**	Device characteristics of 3 wt% of the compounds doped in BH.	45
**Supplementary Table 13**	Device characteristics of 1, 3, 5, 7 wt% of **DPA-BO** doped in BH.	46
**Supplementary Table 14**	Transient EL data of the compounds.	47
**Supplementary Fig. 50**	Transient EL decay curves of the compounds fitted with TTU model.	48
**Supplementary Table 15**	Key performances of recently reported blue OLEDs with CIE$y \leq 0.1$.	49
**Supplementary Table 16**	Cartesian coordinates of the optimized S_0 geometry of **DPA-BO**.	50
**Supplementary Table 17**	Cartesian coordinates of the optimized S_0 geometry of **d-DPA-BO**.	51
**Supplementary Table 18**	Cartesian coordinates of the optimized S_0 geometry of **DPA-d-BO**.	52
**Supplementary Table 19**	Cartesian coordinates of the optimized S_0 geometry of **d-DPA-d-BO**.	53
**Supplementary Table 20**	Cartesian coordinates of the optimized S_1 geometry of **DPA-BO**.	54
**Supplementary Table 21**	Cartesian coordinates of the optimized S_1 geometry of **d-DPA-BO**.	55
**Supplementary Table 22**	Cartesian coordinates of the optimized S_1 geometry of **DPA-d-BO**.	56
**Supplementary Table 23**	Cartesian coordinates of the optimized S_1 geometry of **d-DPA-d-BO**.	57
**Supplementary Table 24**	Cartesian coordinates of the optimized T_1 geometry of **DPA-BO**.	58
**Supplementary Table 25**	Cartesian coordinates of the optimized T_2 geometry of **DPA-BO**.	59
**Supplementary Table 26**	Cartesian coordinates of the optimized geometry of the dimer of DPA-BO at the M06 level.	60

Supplementary References 61−62

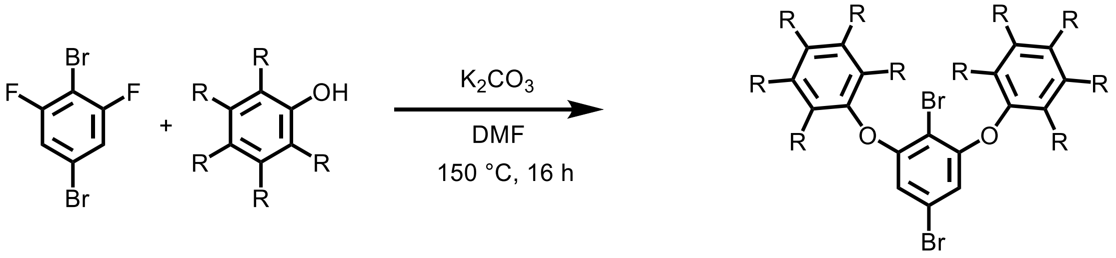

R = H; **2,5-dibromo-1,3-di(phenyloxy)benzene**
R = D; **2,5-dibromo-1,3-di(phenyloxy-*d*$_5$)benzene**

Supplementary Fig. 1 Synthetic route for the BO and d-BO precursor ligands.

2,5-dibromo-1,3-di(phenyloxy)benzene: 2,5-dibromo-1,3-difluorobenzene (6.0 g, 22.07 mmol), phenol (4.16 g, 44.14 mmol), potassium carbonate (12.2 g, 88.28 mmol) and *N*,*N*-dimethylformamide solvent (50 mL) were added into a 250 mL Schlenk tube. With nitrogen filling, the reaction mixture was allowed to react at 150 °C for 16 h. Afterwards, the reaction mixture was diluted with ethyl acetate and poured into water. The aqueous layer was extracted three times with ethyl acetate, then the ethyl acetate layer was collected and dried over anhydrous magnesium sulfate. The organic layer was concentrated under reduced pressure. White powder (7.46 g, 80.5%) was obtained. ^{1}H NMR (400 MHz, Chloroform-*d*) δ 7.45–7.36 (m, 4H), 7.25–7.17 (m, 2H), 7.10–7.03 (m, 4H), 6.76 (s, 2H). High-resolution ESI-MS calcd for $C_{18}H_{12}Br_2O_2$ 419.9184; found 420.9194 ($[M+H]^+$).

2,5-dibromo-1,3-di(phenyloxy-*d*$_5$)benzene: This compound was synthesized by a procedure similar to that of **2,5-dibromo-1,3-di(phenyloxy)benzene,** except that phenol-*d*$_5$ (2.57 g, 25.92 mmol) was used instead of phenol. White powder (3.61 g, 64.7%) was obtained. ^{1}H NMR (400 MHz, Chloroform-*d*) δ 6.76 (s, 2H). High-resolution ESI-MS calcd for $C_{18}H_2D_{10}Br_2O_2$ 429.9811; found 430.9835 ($[M+H]^+$).

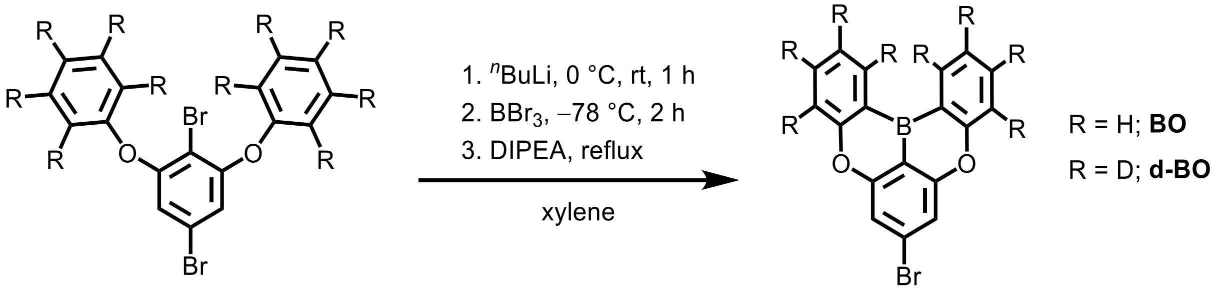

Supplementary Fig. 2 Synthetic route for the BO and d-BO units.

7-bromo-5,9-dioxa-13b-boranaphtho[3,2,1-de]anthracene (BO): 2,5-dibromo-1,3-di(phenyloxy)benzene (2.67 g, 6.36 mmol) in anhydrous xylene (25 mL) was added *n*-butyllithium (7.65 mL, 12.72 mmol) dropwise at 0 °C under a nitrogen atmosphere. After stirring at room temperature for 1 h, BBr_3 (12.72 mL, 1 M) was added at –78 °C. The reaction mixture was allowed to warm to room temperature and stirred for 2 h. *N*,*N*-diisopropylethylamine (3.15 mL, 12.72 mmol) was added at 0 °C and stirred at 130 °C for 12 h. Afterwards, the reaction mixture was diluted with dichloromethane and poured into water. The aqueous layer was extracted three times with dichloromethane, then the dichloromethane layer was collected and dried over anhydrous magnesium sulfate. The organic layer was concentrated under reduced pressure. Yellow oil was purified by silica gel column chromatography (hexane/dichloromethane 5:1 v/v) to afford white powder (0.58 g, 26.0%). ^{1}H NMR (400 MHz, Chloroform-*d*) δ 8.68 (dd, *J* = 7.7, 1.7 Hz, 2H), 7.73 (m, *J* = 8.6, 7.1, 1.7 Hz, 2H), 7.54 (dd, *J* = 8.4, 1.1 Hz, 2H), 7.45–7.37 (m, 4H). High-resolution ESI-MS calcd for $C_{18}H_{10}BBrO_2$ 346.9957; found 347.9980 ([M+H]$^+$).

7-bromo-5,9-dioxa-13b-boranaphtho[3,2,1-de]anthracene-1,2,3,4,10,11,12,13-*d*$_8$ (d-BO): This compound was synthesized by a procedure similar to that of BO, except that 1,1′-((2,5-dibromo-1,3-phenylene)bis(oxy)bis(benzene-2,3,4,5,6-*d*$_5$) (3.61 g, 8.39 mmol) was used instead of 2,5-dibromo-1,3-di(phenyloxy)benzene. White powder (0.73 g, 24.0%) was obtained. ^{1}H NMR (400 MHz, Chloroform-*d*) δ 7.43 (s, 2H). High-resolution ESI-MS calcd for $C_{18}H_2D_8BBrO_2$ 356.0459; found 357.0536 ([M+H]$^+$).

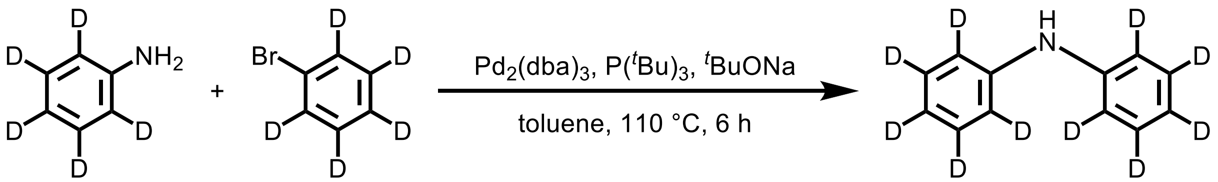

Supplementary Fig. 3 Synthetic route for d-DPA.

Diphenyl-*d*$_{10}$-amine (d-DPA): Aniline-*d*$_5$ (1.82 g, 18.52 mmol), bromobenzene-*d*$_5$ (3 g, 18.52 mmol), sodium *tert*-butoxide (3.56 g, 37.04 mmol), tris(dibenzylideneacetone)dipalladium (0.1 g, 0.93 mmol) and tri-*tert*-butylphosphine (0.04 g, 1.86 mmol) were dissolved in 20 mL of toluene and stirred at 110 °C for 6 h in a 50 mL Schlenk tube. Afterwards, the reaction mixture was diluted with dichloromethane and poured into water. The aqueous layer was extracted three times with dichloromethane, then the dichloromethane layer was collected and dried over anhydrous magnesium sulfate. The organic layer was concentrated under reduced pressure. Yellow oil was purified by silica gel column chromatography (hexane/dichloromethane 4:1 v/v) to afford pale brown powder (3.17 g, 94.5%). ^{1}H NMR (400 MHz, Chloroform-*d*) δ 5.76 (s, 1H). High-resolution ESI-MS m/z calcd for $C_{12}HD_{10}N$ 180.1519, found 180.1590.

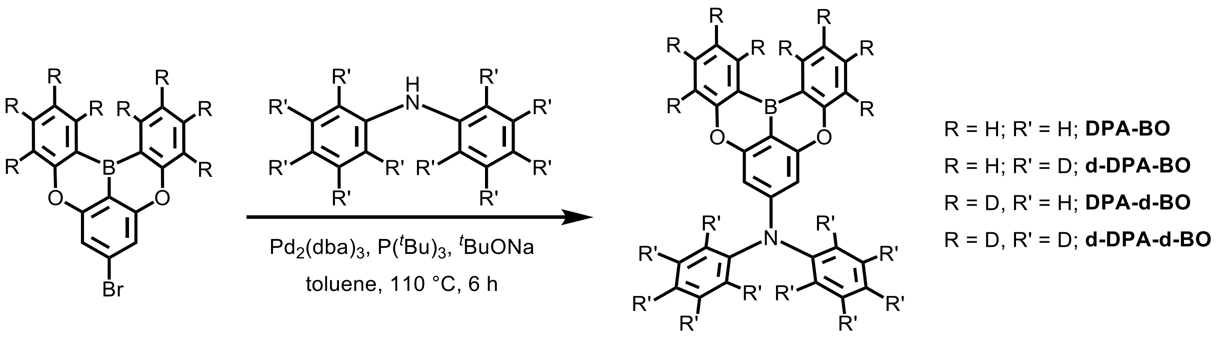

Supplementary Fig. 4 Synthetic route for the TADF compounds.

N,N-diphenyl-5,9-dioxa-13b-boranaphtho[3,2,1-de]anthracen-7-amine (DPA-BO): 7-bromo-5,9-dioxa-13b-boranaphtho[3,2,1-de]anthracene (0.30 g, 0.86 mmol), diphenylamine (0.17 g, 1.03 mmol), sodium *tert*-butoxide (0.17 g, 1.72 mmol), tris(dibenzylideneacetone)dipalladium (0.05 g, 0.05 mmol) and tri-*tert*-butylphosphine (0.02 g, 0.1 mmol) were dissolved in 10 mL of toluene and stirred at 110 °C for 6 h in a 50 mL Schlenk tube. Afterwards, the reaction mixture was diluted with dichloromethane and poured into water. The aqueous layer was extracted three times with dichloromethane, then the dichloromethane layer was collected and dried over anhydrous magnesium sulfate. The organic layer was concentrated under reduced pressure. Yellow powder was purified by silica gel column chromatography (hexane/dichloromethane 6:1 v/v) to afford white powder (0.13 g, 34.9%). ^{1}H NMR (400 MHz, DMSO-d_6) δ 8.68–8.61 (m, 1H), 7.75–7.67 (m, 2H), 7.48 (t, J = 7.8 Hz, 6H), 7.39 (t, J = 7.4 Hz, 2H), 7.36–7.26 (m, 6H), 6.47 (s, 2H). ^{13}C NMR (101 MHz, Chloroform-*d*) δ 160.58, 158.46, 154.28, 146.66, 134.37, 132.99, 129.68, 126.55, 124.88, 122.64, 118.16, 100.20, 77.23. High-resolution ESI-MS m/z calcd for $C_{30}H_{21}BNO_2$ 437.1665, found 438.1647 ([M+H]$^+$).

N,N-bis(phenyl-d_5)-5,9-dioxa-13b-boranaphtho[3,2,1-de]anthracen-7-amine (d-DPA-BO): This compound was synthesized by a procedure similar to that of **DPA-BO**, except that bis(phenyl-d_5)amine (0.14 g, 0.80 mmol) was used instead of diphenylamine. White powder (0.13 g, 17.4%) was obtained. ^{1}H NMR (400 MHz, DMSO-d_6) δ 8.65 (d, J = 7.7 Hz, 1H), 7.71 (t, J = 7.7 Hz, 2H), 7.46 (d, J = 7.8 Hz, 2H), 7.39 (t, J = 7.3 Hz, 2H), 6.48 (s, 2H). ^{13}C NMR (101 MHz, Chloroform-*d*) δ 160.57, 158.45, 154.28, 146.52, 134.36, 132.98, 129.35, 129.16,

128.97, 126.40, 126.28, 126.08, 125.89, 122.63, 118.15, 100.18, 77.22. High-resolution ESI-MS m/z calcd for $C_{30}H_{10}D_{10}BNO_2$ 447.2293, found 448.2269 ([M+H]$^+$).

N,N-diphenyl-5,9-dioxa-13b-boranaphtho[3,2,1-de]anthracen-7-amine-1,2,3,4,10,11,12,13-*d*$_8$ (DPA-d-BO): This compound was synthesized by a procedure similar to that of **DPA-BO**, except that 7-bromo-5,9-dioxa-13b-boranaphtho[3,2,1-de]anthracene-1,2,3,4,10,11,12,13-*d*$_8$ (0.36 g, 1.01 mmol) was used instead of 7-bromo-5,9-dioxa-13b-boranaphtho[3,2,1-de]anthracene. White powder (0.13 g, 28.7%) was obtained. ^{1}H NMR (400 MHz, DMSO-*d*$_6$) δ 7.48 (t, J = 7.7 Hz, 4H), 7.36–7.25 (m, 6H), 6.48 (s, 2H). ^{13}C NMR (101 MHz, Chloroform-*d*) δ 160.55, 158.46, 154.28, 146.66, 129.67, 126.54, 124.87, 122.40, 118.04, 100.21. High-resolution ESI-MS m/z calcd for $C_{30}H_{12}D_8BNO_2$ 445.2167, found 446.2147 ([M+H]$^+$).

N,N-bis(phenyl-*d*$_5$)-5,9-dioxa-13b-boranaphtho[3,2,1-de]anthracen-7-amine-1,2,3,4,10,11,12,13-*d*$_8$ (d-DPA-d-BO): This compound was synthesized by a procedure similar to that of **DPA-BO**, except that 7-bromo-5,9-dioxa-13b-boranaphtho[3,2,1-de]anthracene-1,2,3,4,10,11,12,13-*d*$_8$ (0.36 g, 1.01 mmol) and bis(phenyl-*d*$_5$)amine (0.18 g, 1.01 mmol) were used instead of 7-bromo-5,9-dioxa-13b-boranaphtho[3,2,1-de]anthracene and diphenylamine, respectively. White powder (0.12 g, 25.7%) was obtained. ^{1}H NMR (400 MHz, DMSO-*d*$_6$) δ 6.48 (s, 1H). ^{13}C NMR (101 MHz, Chloroform-*d*) δ 160.54, 158.46, 154.28, 146.52, 129.66, 129.35, 129.16, 128.96, 126.54, 126.08, 125.88, 122.39, 118.02, 100.18. ESI-MS m/z calcd for $C_{30}H_2D_{18}BNO_2$ 455.2795, found 456.2776 ([M+H]$^+$).

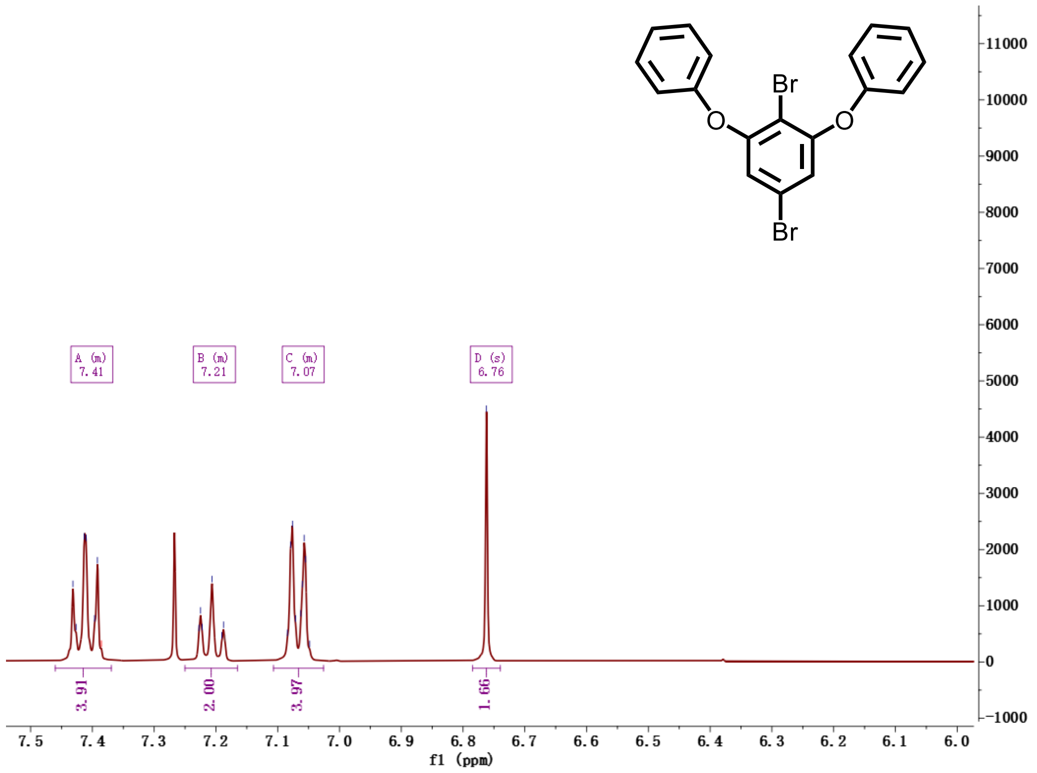

Supplementary Fig. 5 ^{1}H NMR spectrum of 2,5-dibromo-1,3-di(phenyloxy)benzene in $CDCl_3$.

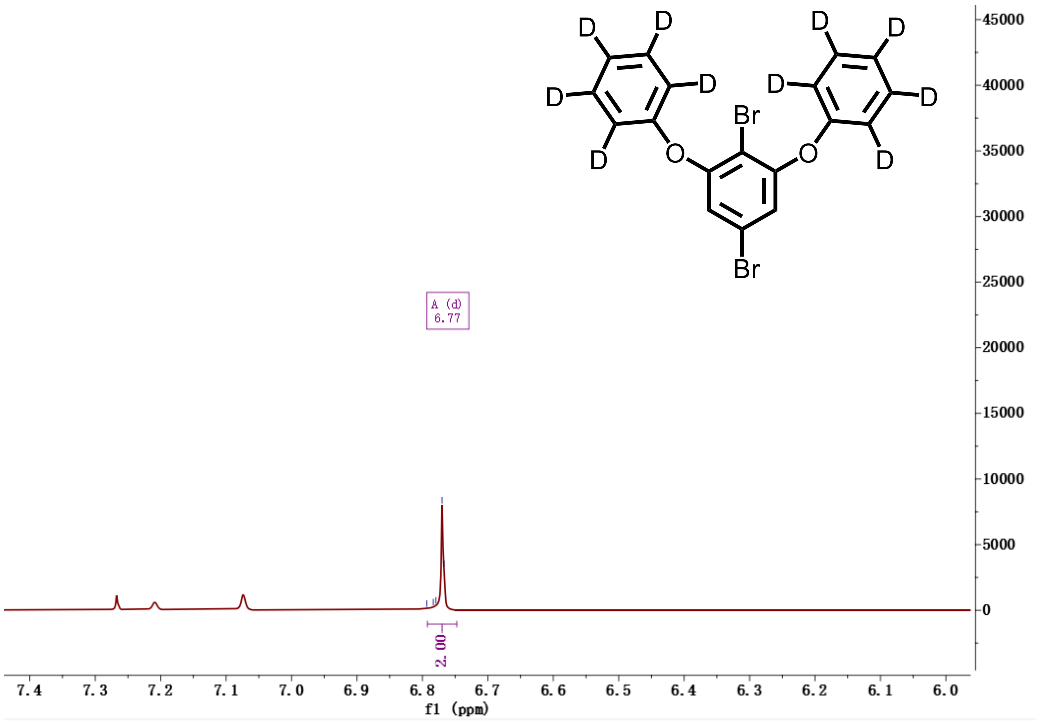

Supplementary Fig. 6 ^{1}H NMR spectrum of 2,5-dibromo-1,3-di(phenyloxy)benzene-d_5 in $CDCl_3$.

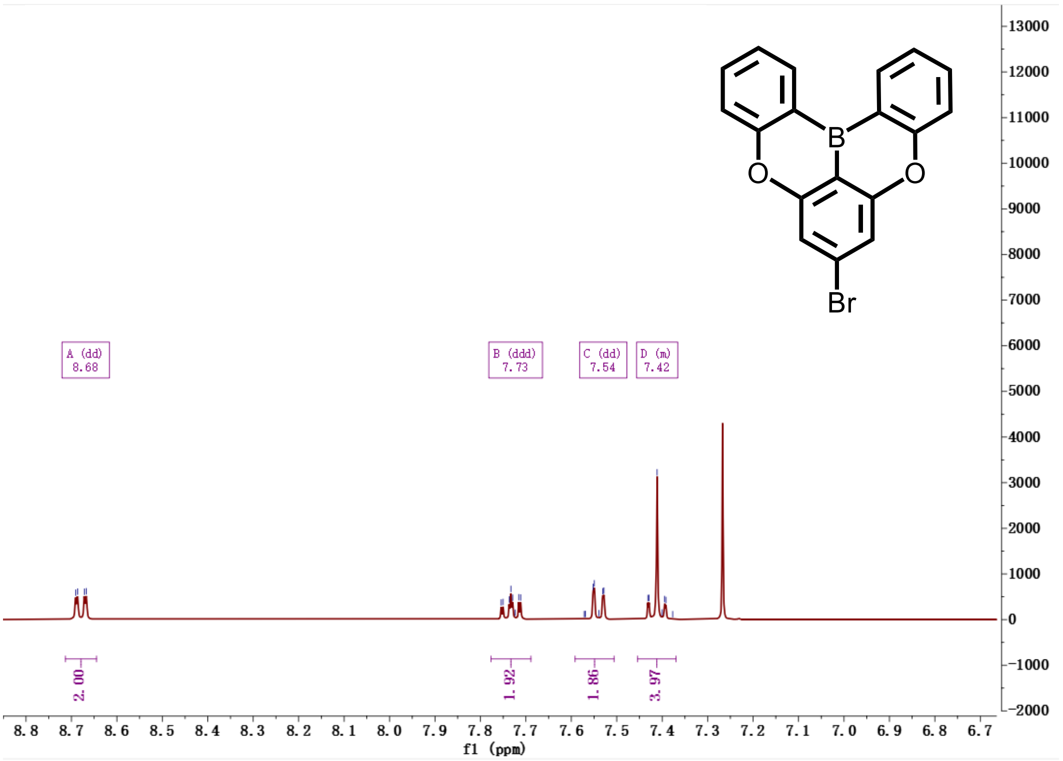

Supplementary Fig. 7 ^{1}H NMR spectrum of BO in $CDCl_3$.

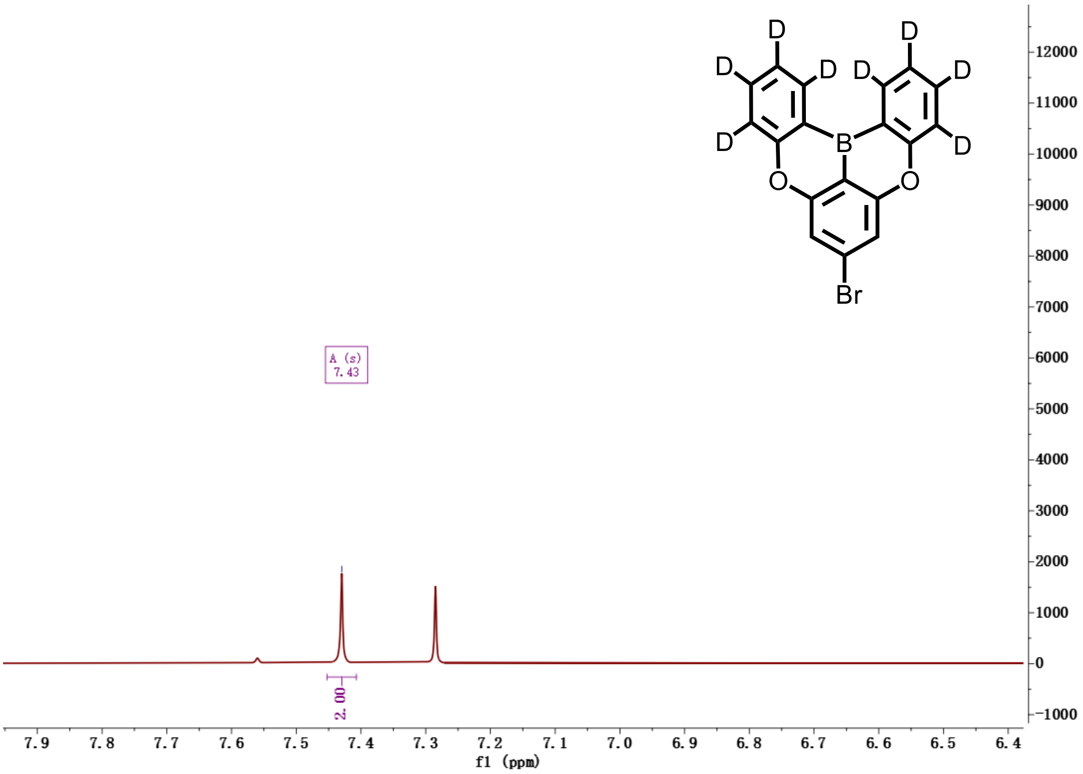

Supplementary Fig. 8 ^{1}H NMR spectrum of d-BO in $CDCl_3$.

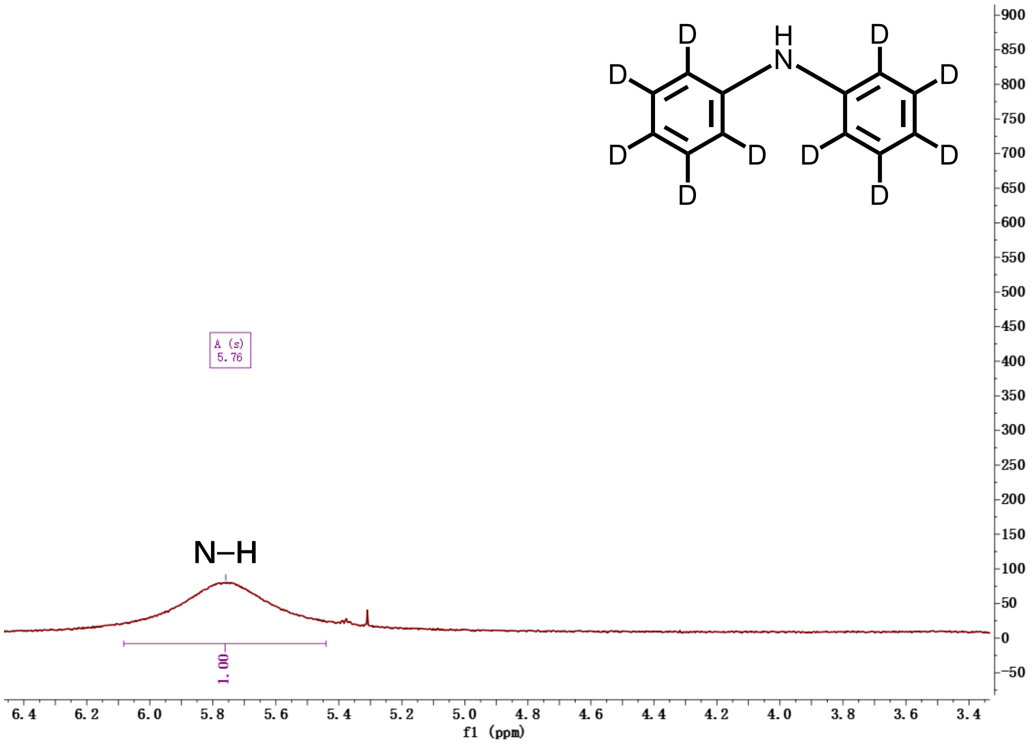

Supplementary Fig. 9 ^{1}H NMR spectrum of d-DPA in $CDCl_3$.

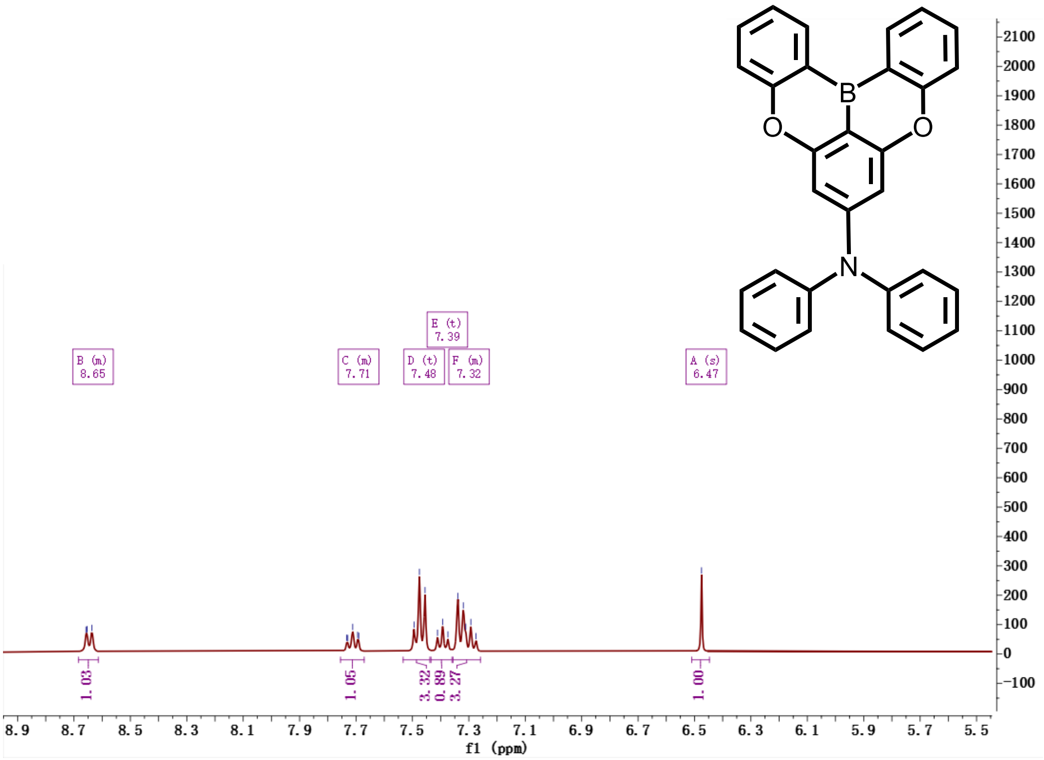

Supplementary Fig. 10 ^{1}H NMR spectrum of **DPA-BO** in DMSO-d_6.

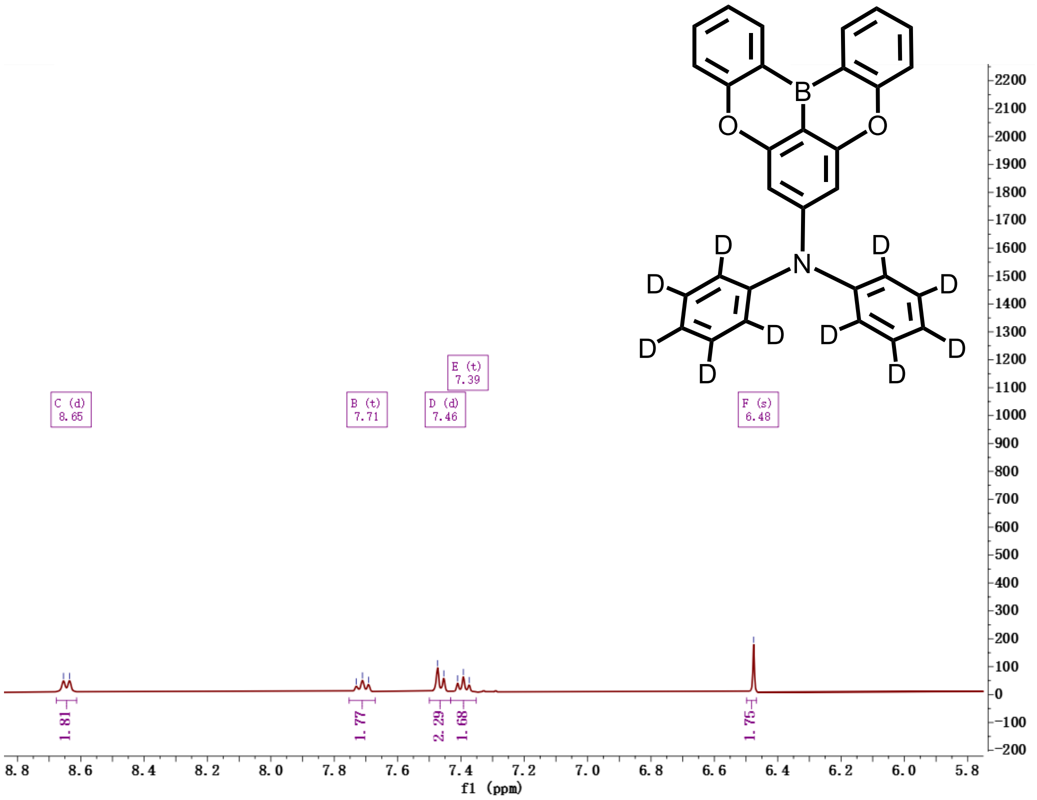

Supplementary Fig. 11 ^{1}H NMR spectrum of **d-DPA-BO** in DMSO-d_6.

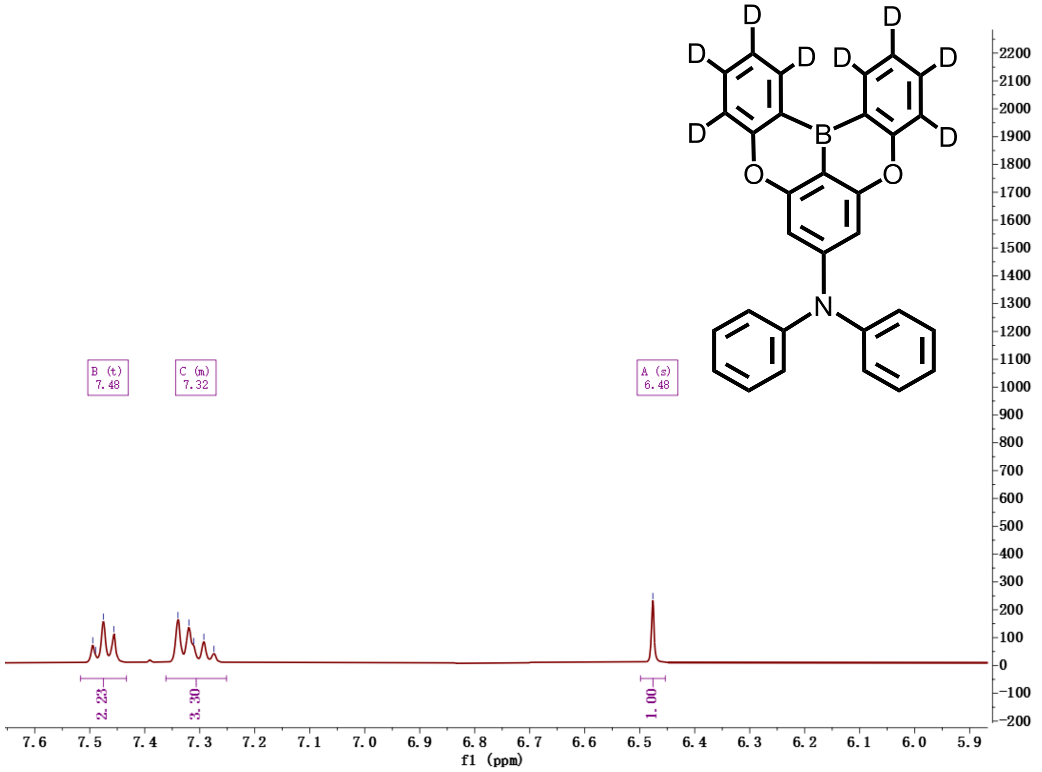

Supplementary Fig. 12 ^{1}H NMR spectrum of **DPA-d-BO** in DMSO-d_6.

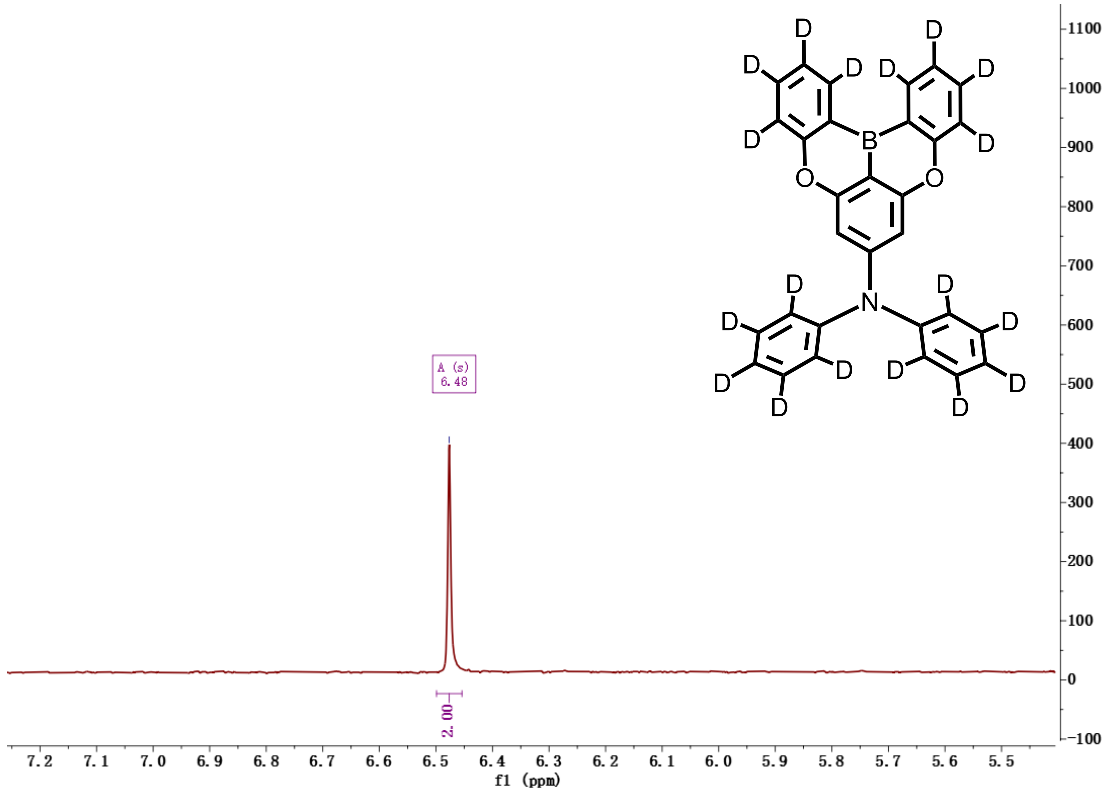

Supplementary Fig. 13 ^{1}H NMR spectrum of **d-DPA-d-BO** in DMSO-d_6.

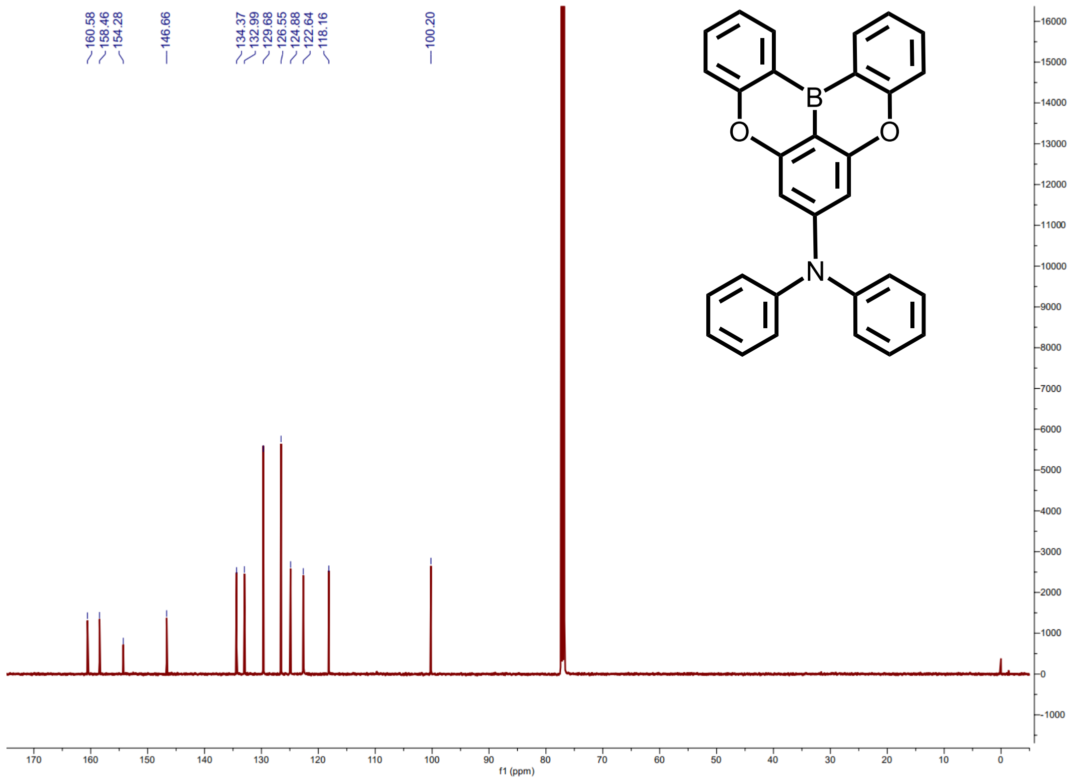

Supplementary Fig. 14 ^{13}C NMR spectrum of **DPA-BO** in $CDCl_3$.

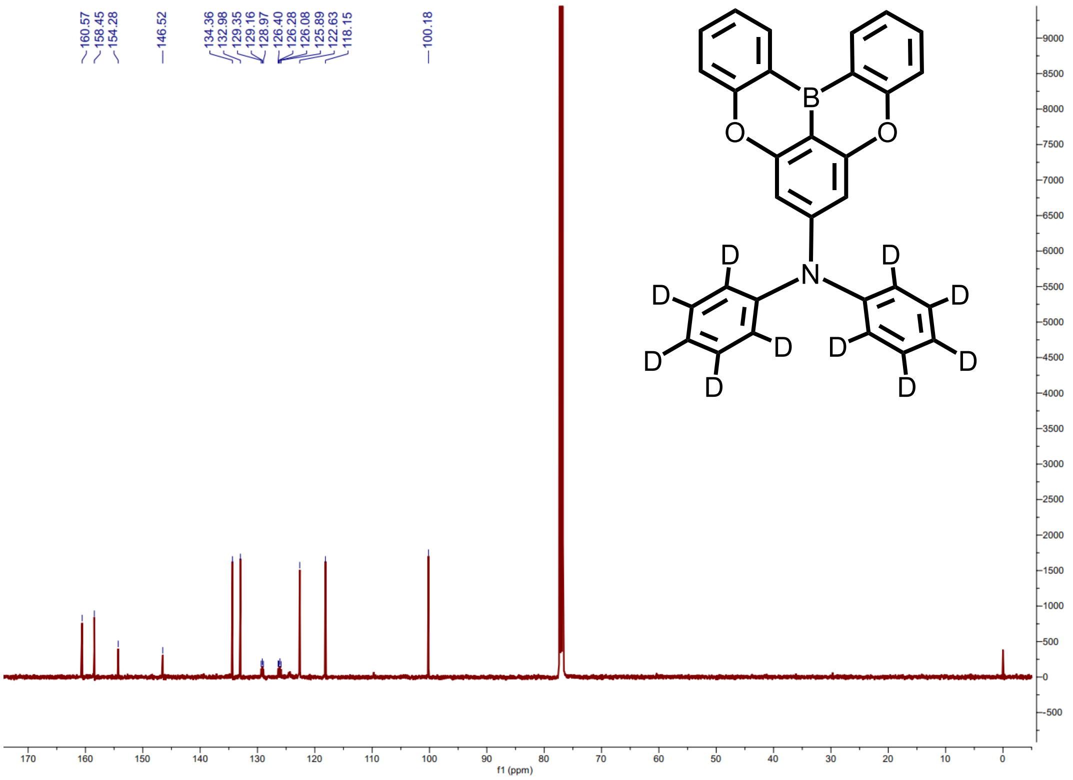

Supplementary Fig. 15 ^{13}C NMR spectrum of **d-DPA-BO** in $CDCl_3$.

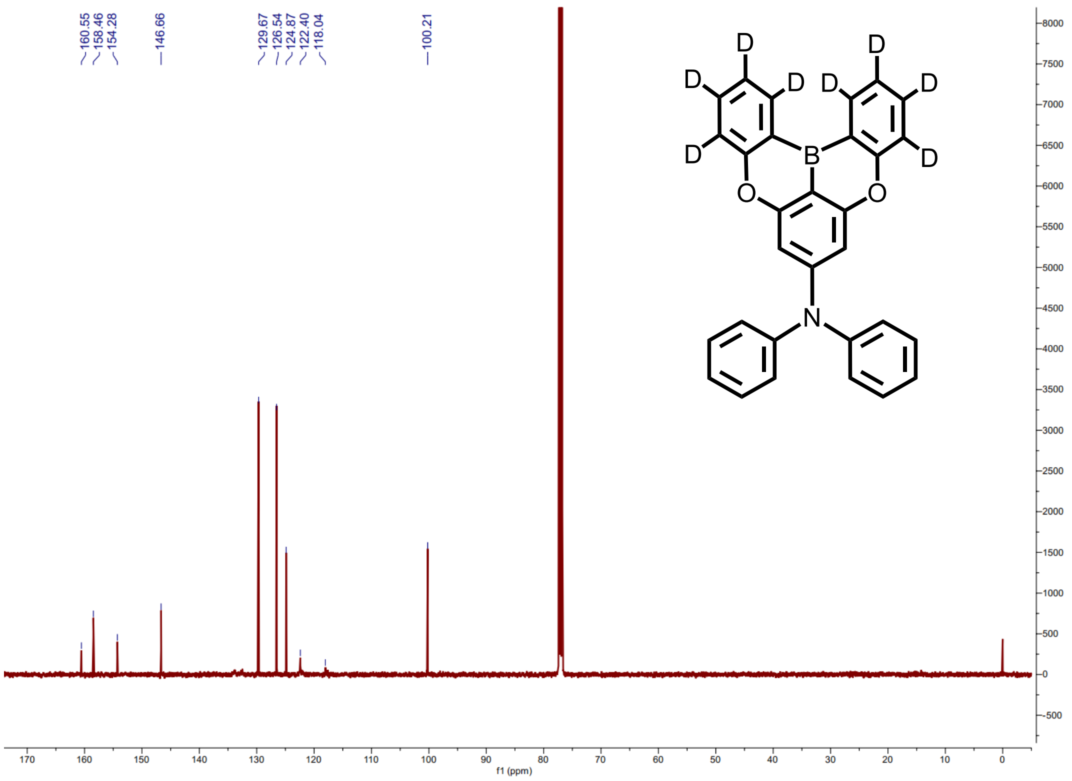

Supplementary Fig. 16 ^{13}C NMR spectrum of **DPA-d-BO** in $CDCl_3$.

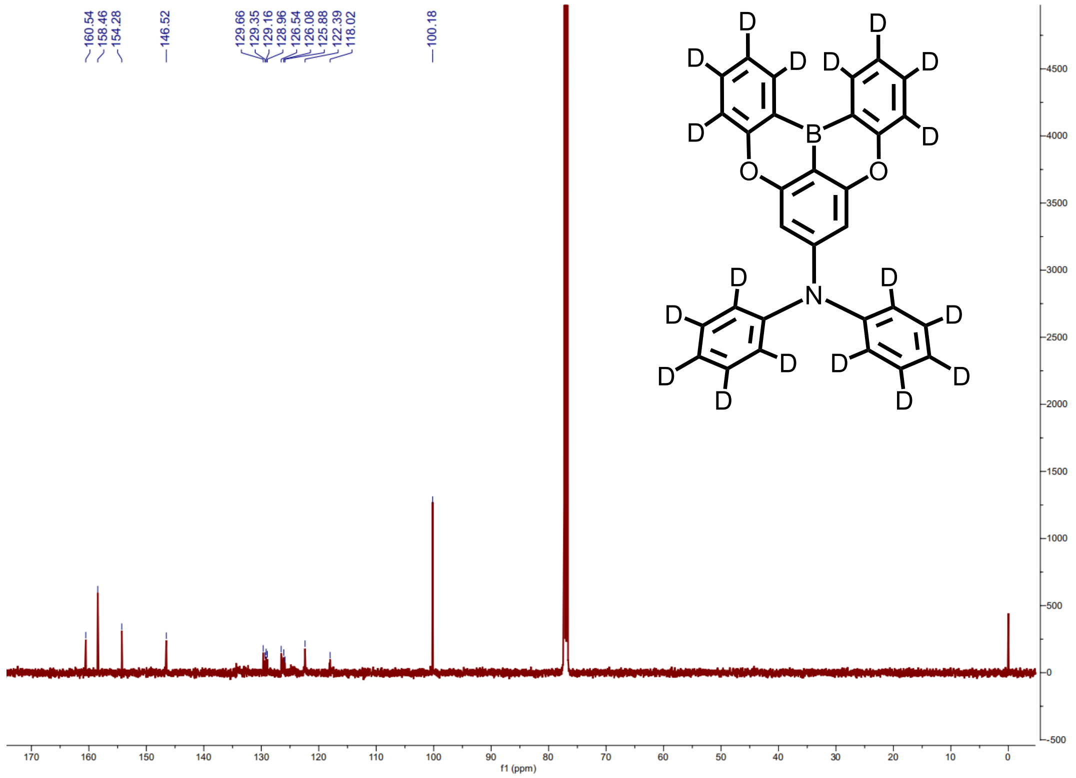

Supplementary Fig. 17 ^{13}C NMR spectrum of **d-DPA-d-BO** in $CDCl_3$.

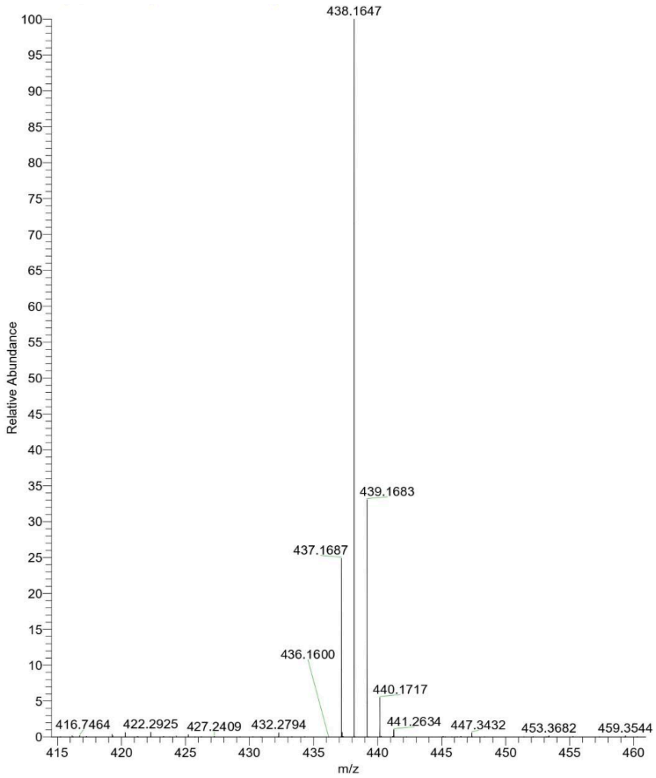

Supplementary Fig. 18 High-resolution ESI-MS of **DPA-BO**.

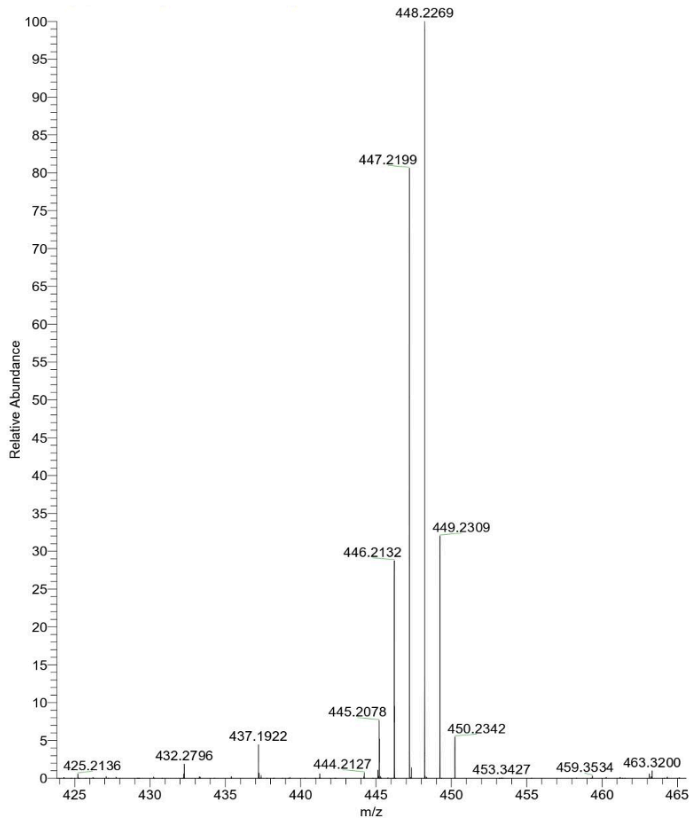

Supplementary Fig. 19 High-resolution ESI-MS of **d-DPA-BO**.

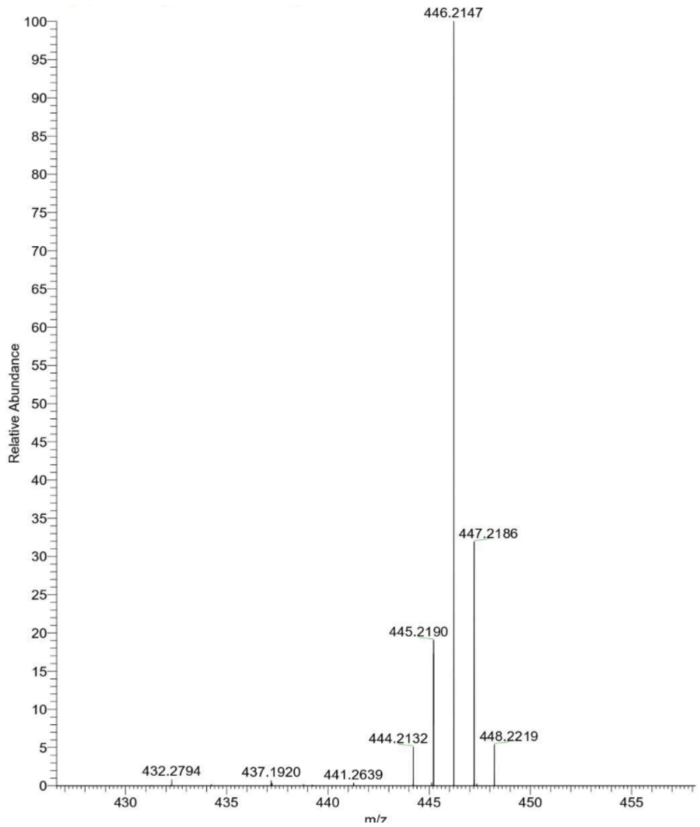

Supplementary Fig. 20 High-resolution ESI-MS of **DPA-d-BO**.

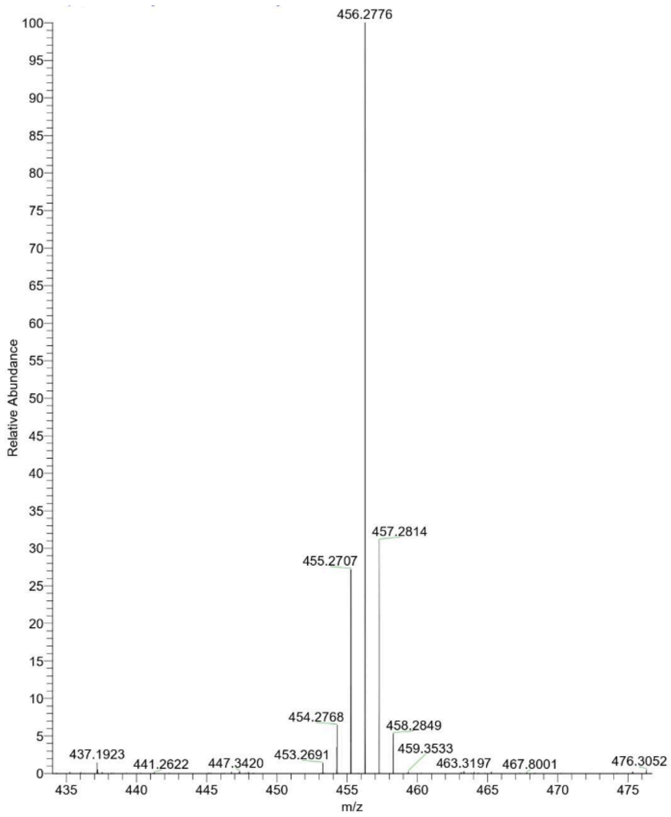

Supplementary Fig. 21 High-resolution ESI-MS of **d-DPA-d-BO**.

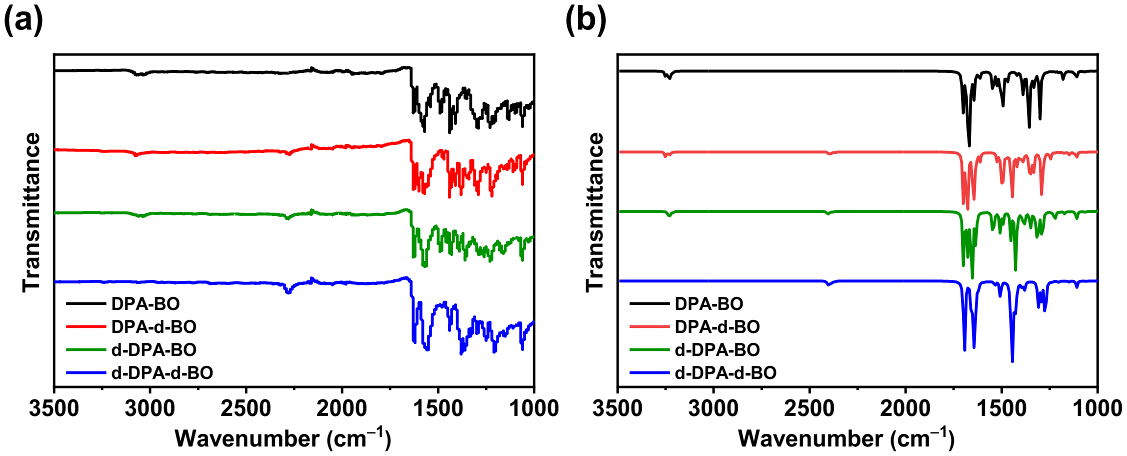

Supplementary Fig. 22 IR spectra of the TADF compounds. (a) Fourier transform stacked spectra (b) simulated stacked spectra.

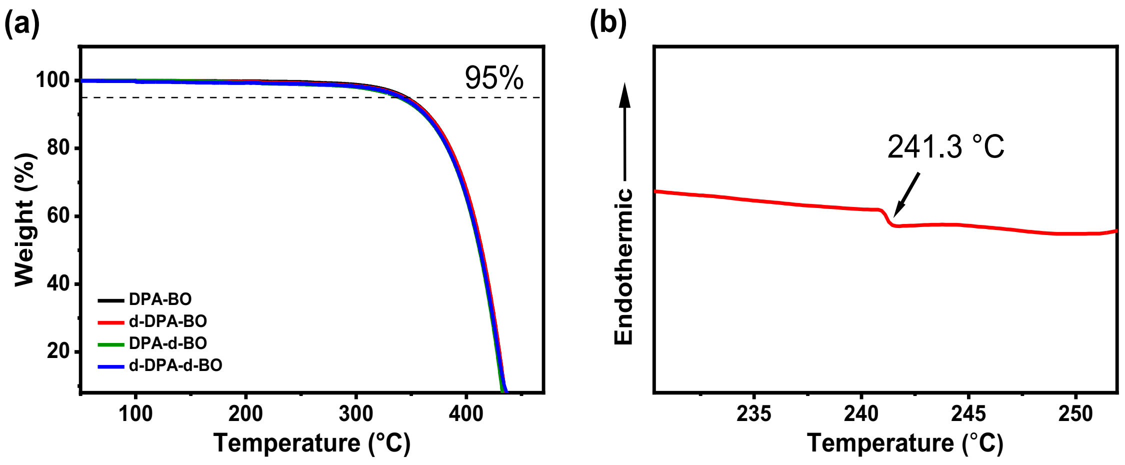

Supplementary Fig. 23 (a) Thermogravimetric analysis traces of the TADF compounds. (b) Differential scanning calorimetry trace of the representative compound **d-DPA-BO** at a heating rate of 10.0 °C min^{-1}.

Supplementary Table 1 Thermal properties of the TADF compounds.

Compound	T_d [a] (ºC)	T_g (ºC)
**DPA-BO**	345.1	258.1
**d-DPA-BO**	343.5	241.3
**DPA-d-BO**	339.1	256.6
**d-DPA-d-BO**	341.3	257.9

[a] Decomposition temperature (T_d) is defined as the temperature at which the compounds show 5 % weight loss.

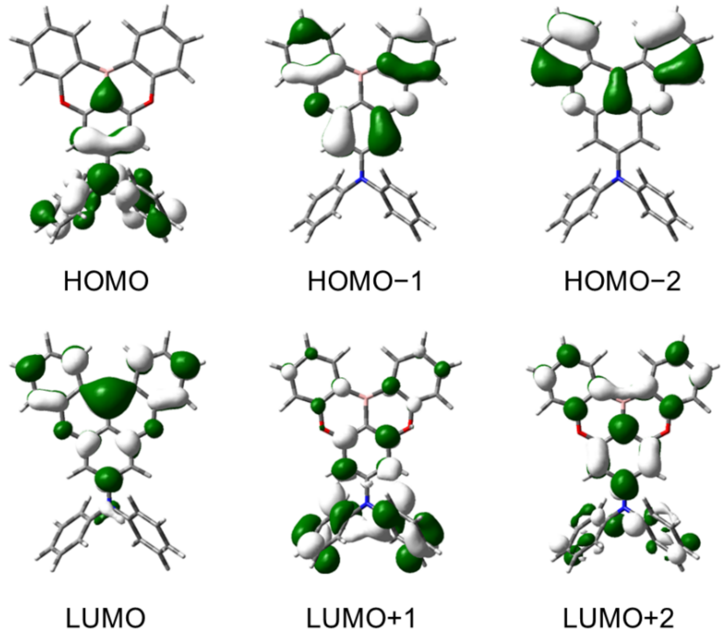

Supplementary Fig. 24 Spatial plots (isovalue = 0.03) of selected molecular orbitals of **DPA-BO** at the optimized ground-state geometry.

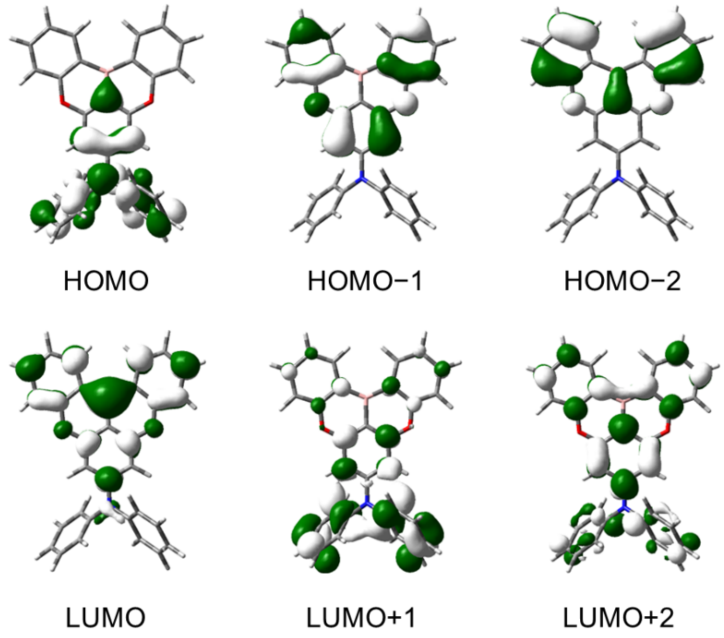

Supplementary Fig. 25 Spatial plots (isovalue = 0.03) of selected molecular orbitals of **d-DPA-BO** at the optimized ground-state geometry.

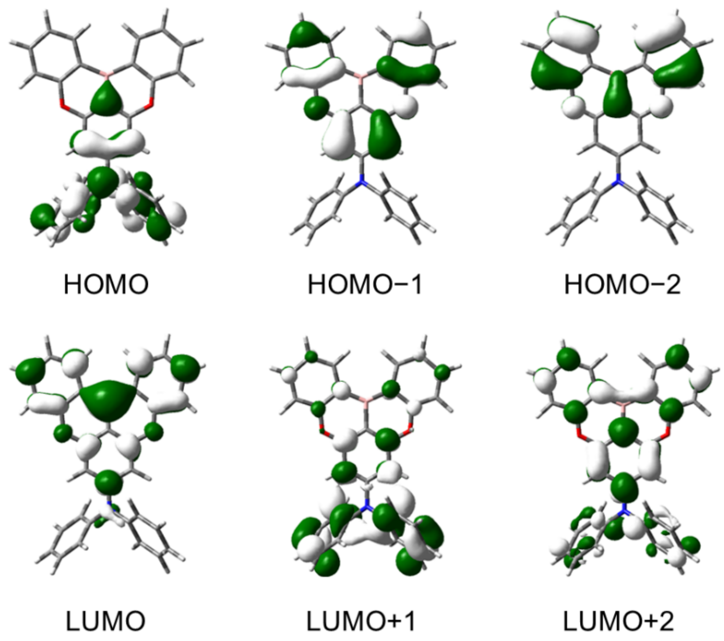

Supplementary Fig. 26 Spatial plots (isovalue = 0.03) of selected molecular orbitals of **DPA-d-BO** at the optimized ground-state geometry.

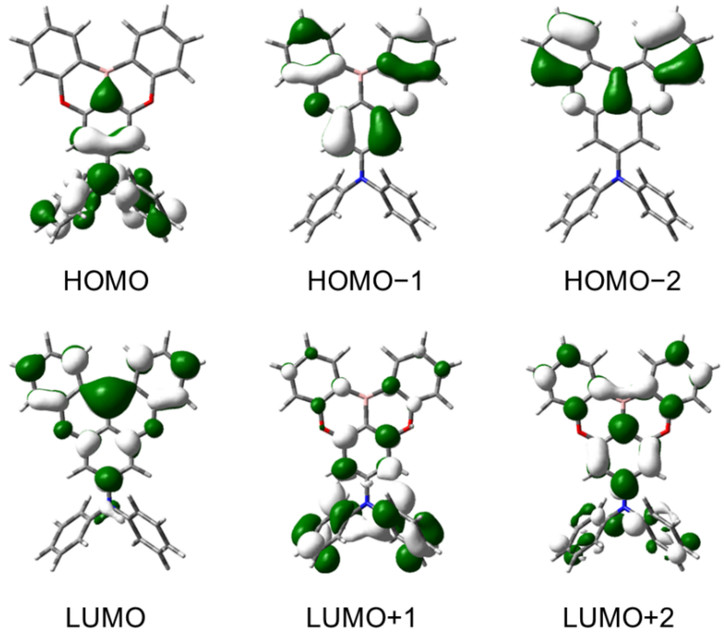

Supplementary Fig. 27 Spatial plots (isovalue = 0.03) of selected molecular orbitals of **d-DPA-d-BO** at the optimized ground-state geometry.

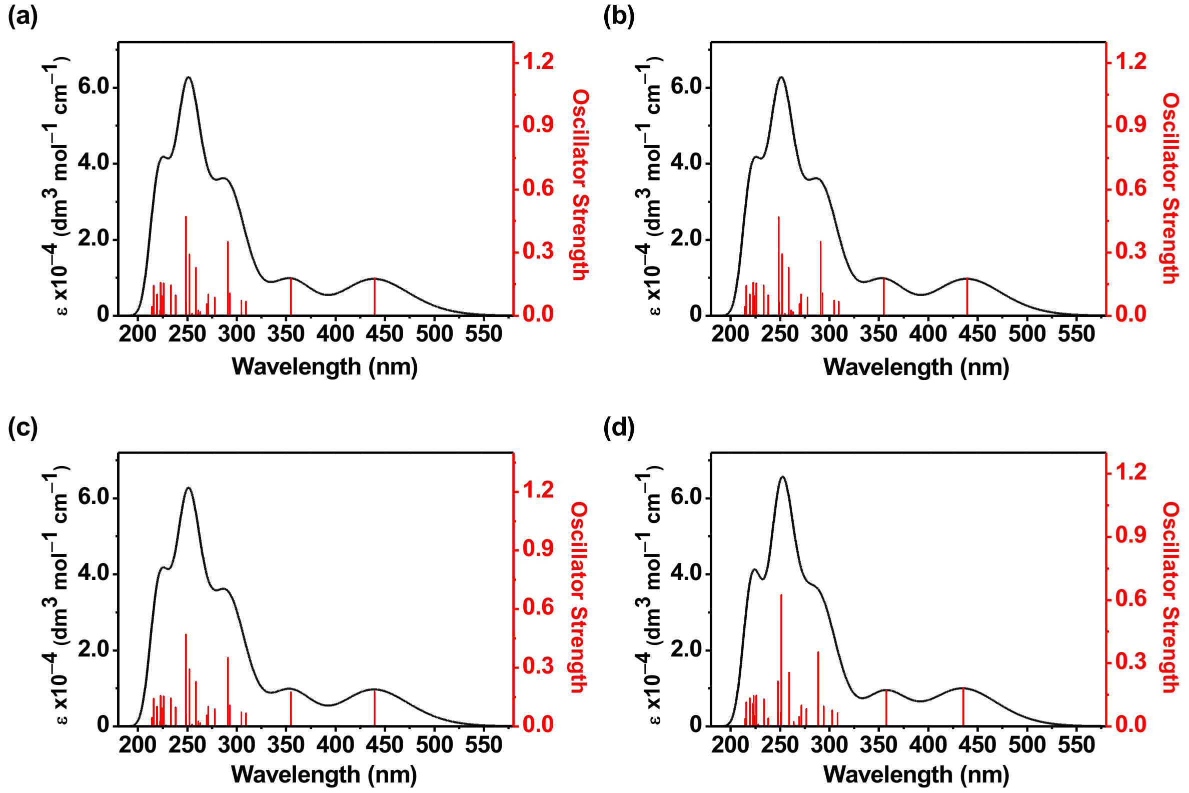

Supplementary Fig. 28 Simulated absorption spectra of (a) **DPA-BO**, (b) **d-DPA-BO**, (c) **DPA-d-BO** and (d) **d-DPA-d-BO** computed by TDDFT/PCM using toluene as the solvent.

Supplementary Table 2 First fifteen singlet excited states (S_n) of the TADF compounds computed by TDDFT/PCM at the PBE0 level using toluene as the solvent.

Compound	S_n	Excitation[a] (coefficient)[b]	Vertical excitation wavelength (nm)	f^c
**DPA-BO**	S_1	H→L (0.70)	377	0.5550
	S_2	H–1→L (0.70)	347	0.1654
	S_3	H→L+1 (0.68)	297	0.0610
	S_4	H→L+3 (0.68)	290	0.2173
	S_5	H–2→L+1 (0.54)	284	0.0227
		H→L+2 (0.43)		
	S_6	H–2→L (0.43)	280	0.2766
		H→L+2 (0.53)		
	S_7	H–1→L+2 (0.44)	270	0.0293
		H→L+4 (0.43)		
	S_8	H→L+6 (0.65)	263	0.0603
	S_9	H–4→L (0.53)	261	0.0109
		H→L+4 (0.36)		
	S_{10}	H–1→L+1 (0.57)	259	0.2496
	S_{11}	H–4→L (0.38)	253	0.3511
		H–4→L+2 (0.45)		
	S_{12}	H–3→L (0.35)	251	0.0718
		H–1→L+1 (0.36)		
		H–1→L+3 (0.41)		
	S_{13}	H–3→L (0.35)	248	0.1039
		H–1→L+1 (0.36)		
		H–1→L+3 (0.44)		
	S_{14}	H–3→L (0.37)	248	0.1446
		H–1→L+3 (0.48)		
	S_{15}	H→L+5 (0.60)	246	0.0133
**d-DPA-BO**	S_1	H→L (0.70)	377	0.5549
	S_2	H–1→L (0.70)	347	0.1654
	S_3	H→L+1 (0.68)	297	0.0609
	S_4	H→L+3 (0.68)	290	0.2174
	S_5	H–2→L+1 (0.54)	284	0.0228
		H→L+2 (0.43)		
	S_6	H–2→L (0.43)	280	0.2765
		H→L+2 (0.53)		
	S_7	H–1→L+2 (0.44)	270	0.0293
		H→L+4 (0.43)		
	S_8	H→L+6 (0.65)	263	0.0603
	S_9	H–4→L (0.53)	261	0.0109
		H→L+4 (0.36)		

	S_{10}	H–1→L+1 (0.57)	259	0.2494
	S_{11}	H–4→L (0.38)	253	0.3509
		H–4→L+2 (0.45)		
	S_{12}	H–3→L (0.35)	251	0.0722
		H–1→L+1 (0.36)		
		H–1→L+3 (0.41)		
	S_{13}	H–3→L (0.35)	248	0.1040
		H–1→L+1 (0.36)		
		H–1→L+3 (0.44)		
	S_{14}	H–3→L (0.37)	248	0.1444
		H–1→L+3 (0.48)		
	S_{15}	H→L+5 (0.60)	246	0.0134
**DPA-d-BO**	S_1	H→L (0.70)	377	0.5550
	S_2	H–1→L (0.70)	347	0.1654
	S_3	H→L+1 (0.68)	297	0.0609
	S_4	H→L+3 (0.68)	290	0.2174
	S_5	H–2→L+1 (0.54)	284	0.0229
		H→L+2 (0.43)		
	S_6	H–2→L (0.43)	280	0.2763
		H→L+2 (0.53)		
	S_7	H–1→L+2 (0.44)	270	0.0294
		H→L+4 (0.43)		
	S_8	H→L+6 (0.65)	263	0.0604
	S_9	H–4→L (0.53)	261	0.0109
		H→L+4 (0.36)		
	S_{10}	H–1→L+1 (0.57)	259	0.2495
	S_{11}	H–4→L (0.38)	253	0.3510
		H–4→L+2 (0.45)		
	S_{12}	H–3→L (0.35)	251	0.0722
		H–1→L+1 (0.36)		
		H–1→L+3 (0.41)		
	S_{13}	H–3→L (0.35)	248	0.1040
		H–1→L+1 (0.36)		
		H–1→L+3 (0.44)		
	S_{14}	H–3→L (0.37)	248	0.1444
		H–1→L+3 (0.48)		
	S_{15}	H→L+5 (0.60)	246	0.0134
**d-DPA-d-BO**	S_1	H→L (0.70)	377	0.5549
	S_2	H–1→L (0.70)	347	0.1654
	S_3	H→L+1 (0.68)	297	0.0608
	S_4	H→L+3 (0.68)	290	0.2175
	S_5	H–2→L+1 (0.54)	284	0.0229

	H→L+2 (0.43)		
S_6	H–2→L (0.43)	280	0.2763
	H→L+2 (0.53)		
S_7	H–1→L+2 (0.44)	270	0.0294
	H→L+4 (0.43)		
S_8	H→L+6 (0.65)	263	0.0604
S_9	H–4→L (0.53)	261	0.0109
	H→L+4 (0.36)		
S_{10}	H–1→L+1 (0.57)	259	0.2494
S_{11}	H–4→L (0.38)	253	0.3510
	H–4→L+2 (0.45)		
S_{12}	H–3→L (0.35)	251	0.0725
	H–1→L+1 (0.36)		
	H–1→L+3 (0.41)		
S_{13}	H–3→L (0.35)	248	0.1040
	H–1→L+1 (0.36)		
	H–1→L+3 (0.44)		
S_{14}	H–3→L (0.37)	248	0.1442
	H–1→L+3 (0.48)		
S_{15}	H→L+5 (0.60)	246	0.0133

[a)] Orbitals involved in the major excitation (H = HOMO and L = LUMO).

[b)] The coefficients in the configuration interaction (CI) expansion.

[c)] Oscillator strengths.

Supplementary Table 3 Relative energies of the lowest-lying singlet excited states (S_1) of the TADF compounds optimized at the PBE0 level.

Compound	$\Delta E(S_1–S_0)/cm^{-1}$ (λ/nm)[a]
**DPA-BO**	24496 (408)
**d-DPA-BO**	24496 (408)
**DPA-d-BO**	24496 (408)
**d-DPA-d-BO**	24496 (408)

[a)] Energy difference between the lowest-lying singlet excited state (S_1) and the ground-state (S_0) at the corresponding optimized geometries in toluene solution.

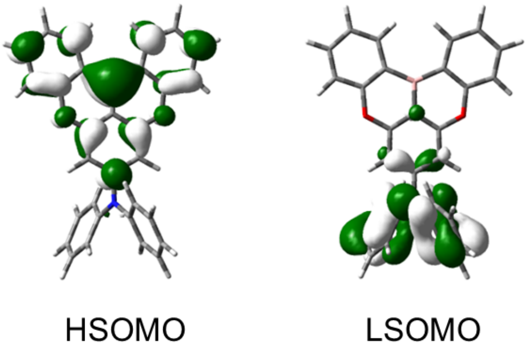

Supplementary Fig. 29 Spatial plots (isovalue = 0.03) of selected molecular orbitals of **DPA-BO** at the optimized S_1 state geometry.

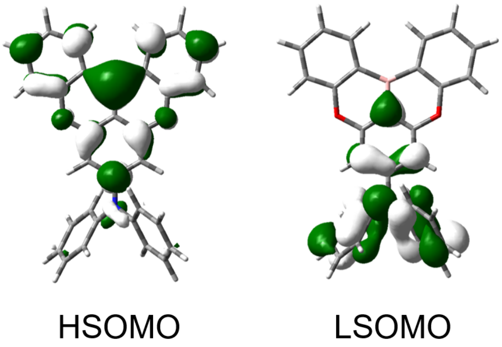

Supplementary Fig. 30 Spatial plots (isovalue = 0.03) of selected molecular orbitals of **DPA-BO** at the optimized T_1 state geometry.

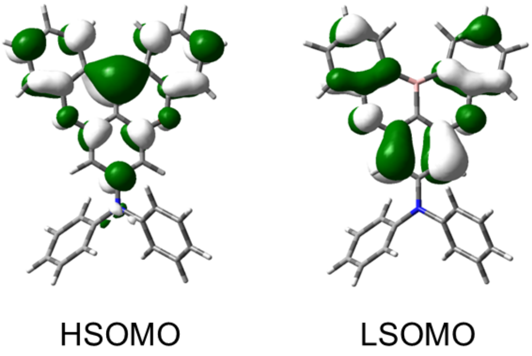

Supplementary Fig. 31 Spatial plots (isovalue = 0.03) of selected molecular orbitals of **DPA-BO** at the optimized T_2 state geometry.

Supplementary Table 4 Zero point energy (ZPE) of the compounds at the ground-state geometry optimized at the PBE0/6-31G(d,p) level.

Compound	ZPE (kcal mol^{-1})
**DPA**	115.2
**d-DPA**	94.4
**BO**	144.0
**d-BO**	127.7
**DPA-BO**	262.9
**d-DPA-BO**	242.5
**DPA-d-BO**	246.6
**d-DPA-d-BO**	226.1

Supplementary Table 5 Bond dissociation energy (BDE) of the C−N bonds of the compounds optimized at the M06-2X/6-311++G(d,p) level.

Compound	BDE (eV)	
	S_0	T_1
**DPA-BO**	3.684	0.663
**d-DPA-BO**	3.684	0.668
**DPA-d-BO**	3.518	0.659
**d-DPA-d-BO**	3.519	0.662

Supplementary Table 6 Bond dissociation energy (BDE) of the C−H or C−D bonds of the compounds optimized at the M06-2X/6-311++G(d,p) level.

Bond	BDE (eV)
C−H bond of **DPA** in **DPA-BO**	4.78
C−D bond of **DPA** in **d-DPA-BO**	4.87
C−H bond of **BO** in **DPA-BO**	4.86
C−D bond of **BO** in **DPA-d-BO**	4.95

Supplementary Table 7 Enthalpies of activation corresponding to the transition state of the C–N bond dissociation of **DPA-BO** and **d-DPA-d-BO** on the triplet potential energy surface.

Compound	Enthalpy of activation (eV)
**DPA-BO**	0.903
**d-DPA-d-BO**	0.904

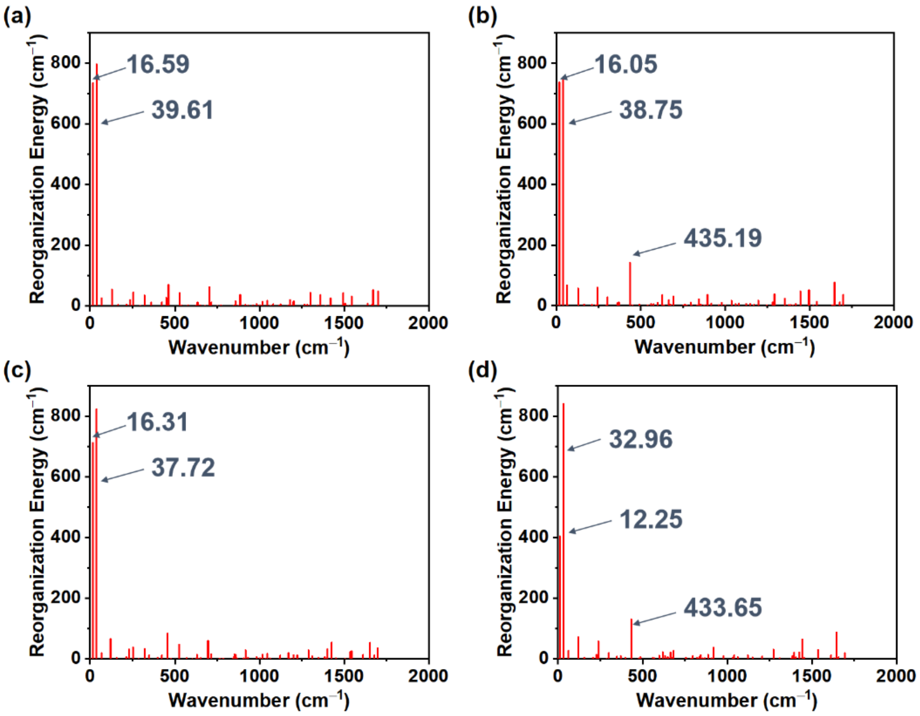

Supplementary Fig. 32 Plots of computed reorganization energies as a function of normal mode wavenumbers for S_1-S_0 of (a) **DPA-BO**, (b) **d-DPA-BO**, (c) **DPA-d-BO** and (d) **d-DPA-d-BO**.

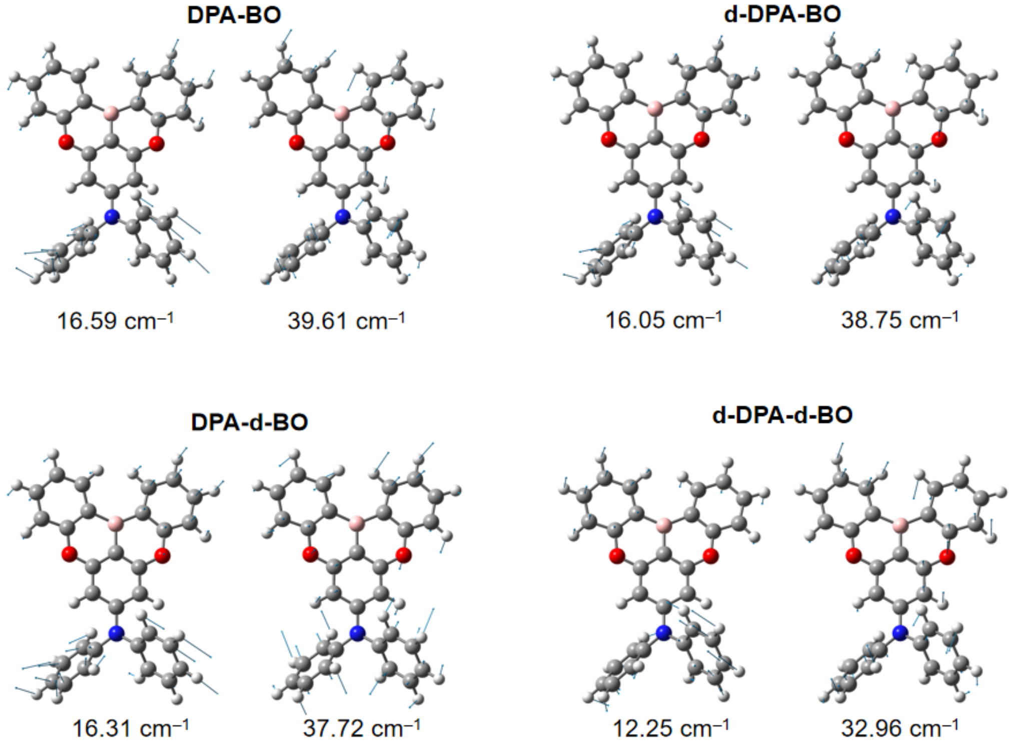

Supplementary Fig. 33 Illustration of selected normal modes contributing to large reorganization energies for the S_0 state.

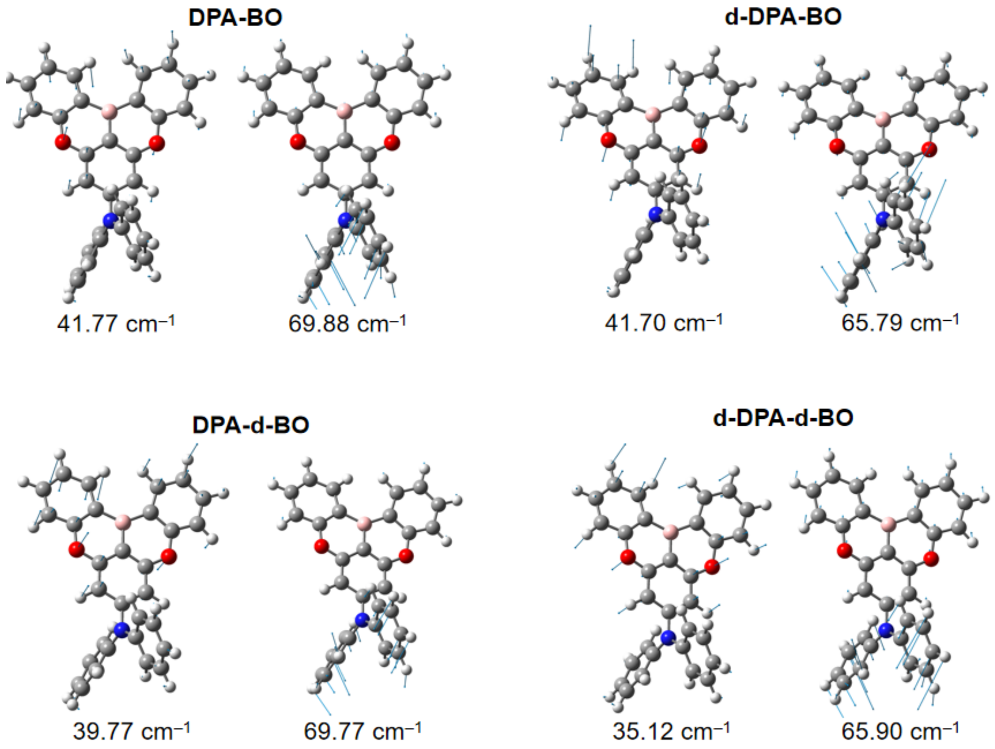

Supplementary Fig. 34 Illustration of selected normal modes contributing to large reorganization energies for the S_1 state.

Supplementary Table 8 Vibrational frequencies (ω_j), displacement (ΔQ), Huang-Rhys factors (S_j) and reorganization energies (λ_j) of selected normal modes for S_0 of the compounds in toluene.

**DPA-BO**				**d-DPA-BO**			
ω_j (cm^{-1})	ΔQ	S_j	λ_j (meV)	ω_j (cm^{-1})	ΔQ	S_j	λ_j (meV)
16.59	-1082.23	44.26	91.02	16.05	-1120.44	45.90	91.35
39.61	-471.75	20.08	98.61	38.75	-468.79	19.40	93.19
462.01	11.87	0.15	8.49	62.81	-86.23	1.06	8.29
704.51	-7.43	0.09	7.74	435.19	-18.12	0.33	17.56
1671.31	-2.85	0.03	6.40	1648.17	3.49	0.05	9.35
**DPA-d-BO**				**d-DPA-d-BO**			
ω_j (cm^{-1})	ΔQ	S_j	λ_j (meV)	ω_j (cm^{-1})	ΔQ	S_j	λ_j (meV)
16.31	1083.23	43.61	88.21	12.25	1086.04	32.93	50.02
37.72	503.86	21.82	102.04	32.96	-582.53	25.48	104.15
121.20	43.98	0.53	8.03	120.36	-46.74	0.60	8.94
455.68	13.30	0.18	10.39	433.65	17.43	0.30	16.14
696.23	-7.36	0.09	7.41	1645.92	3.76	0.05	10.80

Supplementary Table 9 Vibrational frequencies (ω_j), displacement (ΔQ), Huang-Rhys factors (S_j) and reorganization energies (λ_j) of selected normal modes for S_1 of the compounds in toluene.

**DPA-BO**				**d-DPA-BO**			
ω_j (cm^{-1})	ΔQ	S_j	λ_j (meV)	ω_j (cm^{-1})	ΔQ	S_j	λ_j (meV)
11.77	-1062.75	30.28	44.19	11.42	-1093.10	31.10	44.05
41.77	-402.03	15.38	79.64	41.70	-408.05	15.82	81.78
69.88	199.27	6.32	54.77	65.79	-218.53	7.16	58.39
94.66	133.99	3.87	45.44	86.90	-136.34	3.68	39.65
250.63	36.98	0.78	24.26	368.94	22.31	0.42	19.14
**DPA-d-BO**				**d-DPA-d-BO**			
ω_j (cm^{-1})	ΔQ	S_j	λ_j (meV)	ω_j (cm^{-1})	ΔQ	S_j	λ_j (meV)
11.35	1070.12	29.61	41.66	10.56	1085.17	28.32	37.06
39.77	420.11	15.99	78.84	35.12	-455.35	16.59	72.25
69.77	201.09	6.43	55.59	65.90	-246.95	9.16	74.80
94.54	135.00	3.93	46.01	86.16	139.34	3.81	40.71
250.02	-36.44	0.76	23.44	368.86	-22.95	0.44	20.25

Supplementary Table 10 The computed radiative (k_r) and nonradiative (k_{nr}) decay rate constants of the S_1 state.

Compound	k_r (s^{-1})	k_{nr} (s^{-1})
**DPA-BO**	1.18×10^8	1.43×10^{11}
**d-DPA-BO**	1.18×10^8	1.45×10^{11}
**DPA-d-BO**	1.18×10^8	1.44×10^{11}
**d-DPA-d-BO**	1.19×10^8	1.48×10^{11}

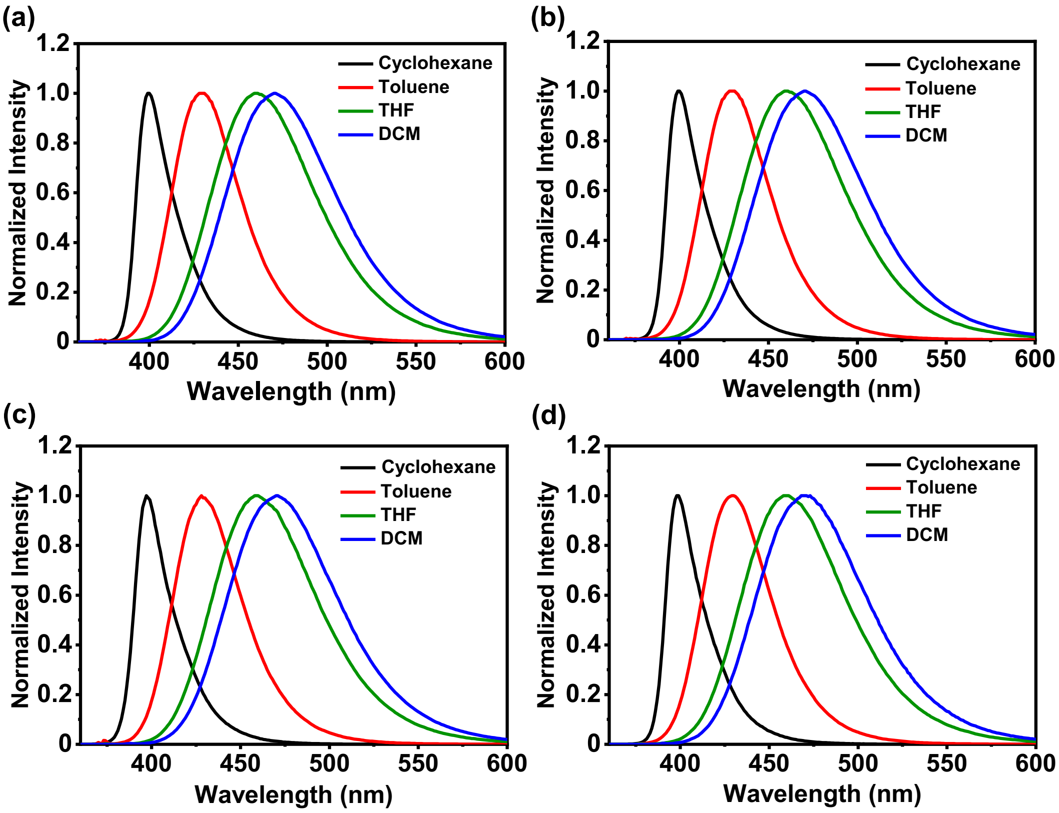

Supplementary Fig. 35 Normalized PL spectra of (a) **DPA-BO**, (b) **d-DPA-BO**, (c) **DPA-d-BO** and (d) **d-DPA-d-BO** in various solvent.

Supplementary Table 11 Photophysical data of the TADF compounds.

Compound	Medium (T [K])	Absorption λ_{max} [nm] (ε_{max}[$dm^3mol^{-1}cm^{-1}$])	Emission λ_{max} [nm] τ_p (ns)/τ_d (μs)[a]	Φ_{PL} (sol)[b]	Φ_{PL} (film)[c]	HOMO (eV)[d]	LUMO (eV)[e]	E_S/E_T (eV)[f]	ΔE_{ST} (eV)[g]
**DPA-BO**	Toluene (298)	296 (10860), 304 (11890), 375 (36210)	430 (4.4)	0.92					
	Cyclohexane (298)		400						
	THF (298)		461						
	DCM (298)		471						
	DPEPO thin film								
	5 wt% (298)		446 (3.2/78.1)		0.93	−5.99	−3.01		
	10 wt% (298)		451 (3.5/67.2)		0.89			3.01	0.08
	15 wt% (298)		455 (3.9/67.3)		0.89				
	20 wt% (298)		458 (4.0/65.5)		0.79				
	10 wt% (77)		450 (5.0/15.2)					2.93	
	mCBP thin film								
	15 wt% with 10 wt% BN3 (298)		569 (4.4/196.3)		0.48				
**d-DPA-BO**	Toluene (298)	296 (10090), 304 (11550), 375 (37790)	430 (4.5)	0.94					
	Cyclohexane (298)		400						
	THF (298)		460						
	DCM (298)		471						
	DPEPO thin film								
	10 wt% (298)		451 (3.4/65.4)			−5.96	−2.96	3.01	0.08
	10 wt% (77)		452 (4.8/11.9)					2.93	
	mCBP thin film								
	15 wt% with 10 wt% BN3 (298)		569 (4.6/174.2)		0.49				

**DPA-d-BO**	Toluene (298)	296 (11150), 304 (12760), 375 (40035)	428 (4.3)	0.96					
	Cyclohexane (298)		398						
	THF (298)		469						
	DCM (298)		471						
	DPEPO thin film								
	10 wt% (298)		449 (3.4/64.1)			−5.96	−2.96	3.01	0.08
	10 wt% (77)		449 (5.3/14.7)					2.93	
	mCBP thin film								
	15 wt% with 10 wt% BN3 (298)		569 (4.7/79.4)		0.57				
**d-DPA-d-BO**	Toluene (298)	296 (10210), 304 (10835), 375 (36500)	430 (4.3)	0.95					
	Cyclohexane (298)		399						
	THF (298)		460						
	DCM (298)		470						
	DPEPO thin film								
	10 wt% (298)		450 (3.4/59.5)			−5.96	−2.96	3.02	0.09
	10 wt% (77)		451 (5.4/11.7)					2.93	
	mCBP thin film								
	15 wt% with 10 wt% BN3 (298)		569 (4.5/134.0)		0.51				

[a)] PL lifetimes of prompt (τ_p) and delayed (τ_d) decay components.

[b)] Measured in oxygen-free toluene at room temperature (298 K).

[c)] Measured at various doping concentrations in DPEPO that were excited at wavelength 374 nm.

[d)] Deduced from the HOMO and optical energy gap (E_g).

[e)] Determined by ultraviolet photo-electron spectroscopy (UPS).

[f)] Singlet (E_s) and triplet (E_T) energies estimated from onsets of the emission spectra at 298 K and 77 K in 10 wt% DPEPO doped films, respectively.

[g)] $\Delta E_{ST} = E_S - E_T$

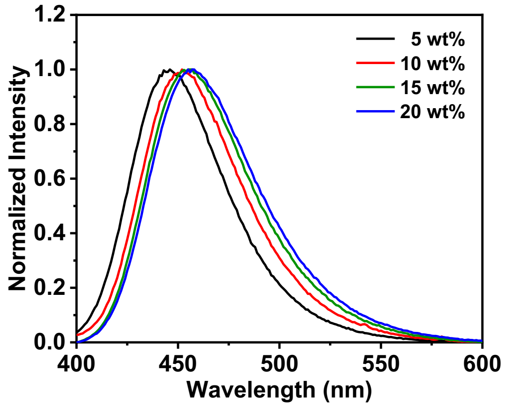

Supplementary Fig. 36 Normalized PL spectra of 5−20 wt% **DPA-BO** doped in DPEPO films at 298 K.

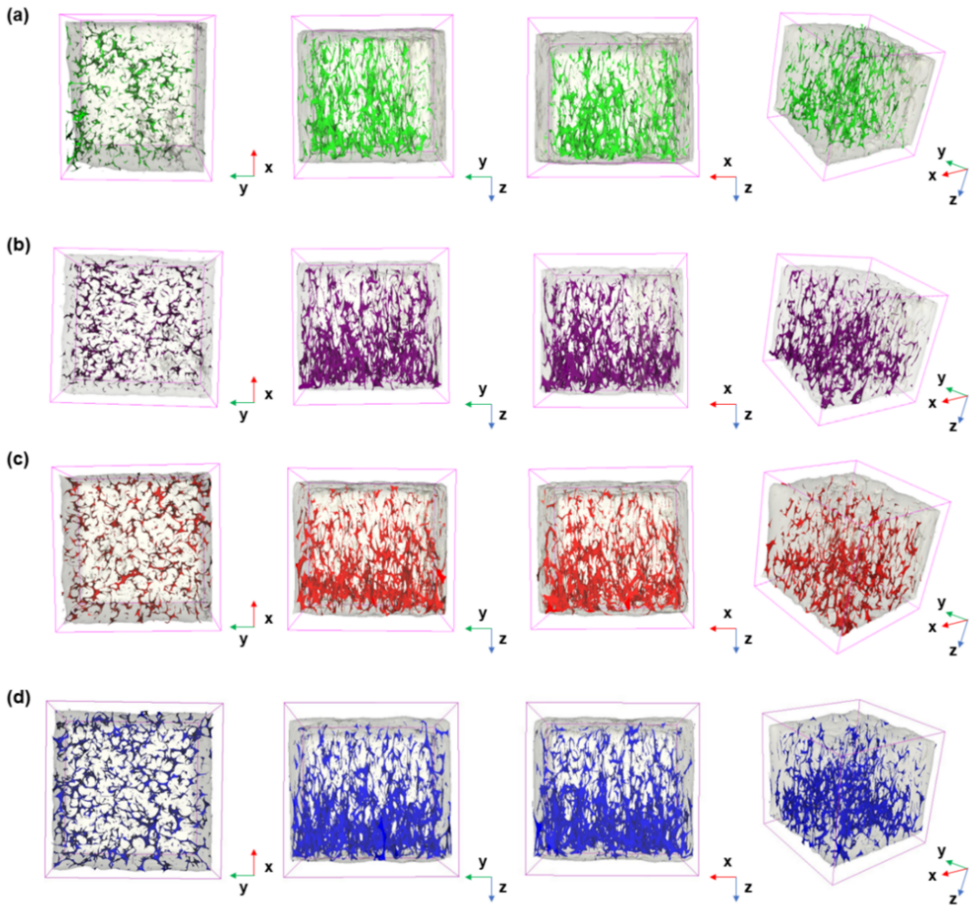

Supplementary Fig. 37. Spatial localization of 10 wt% (a) **DPA-BO** ($C_{30}H_{20}BNO_2^+$, molar mass = 437.16 g/mol), (b) **d-DPA-BO** ($C_{30}H_{10}D_{10}BNO_2^+$, molar mass = 447.23 g/mol), (c) **DPA-d-BO** ($C_{30}H_{12}D_8BNO_2^+$, molar mass = 445.21 g/mol) and (d) **d-DPA-d-BO** ($C_{30}H_2D_{18}BNO_2^+$, molar mass = 455.28 g/mol) in DPEPO ($C_{36}H_{28}O_3P_2^+$, molar mass = 570.09 g/mol).

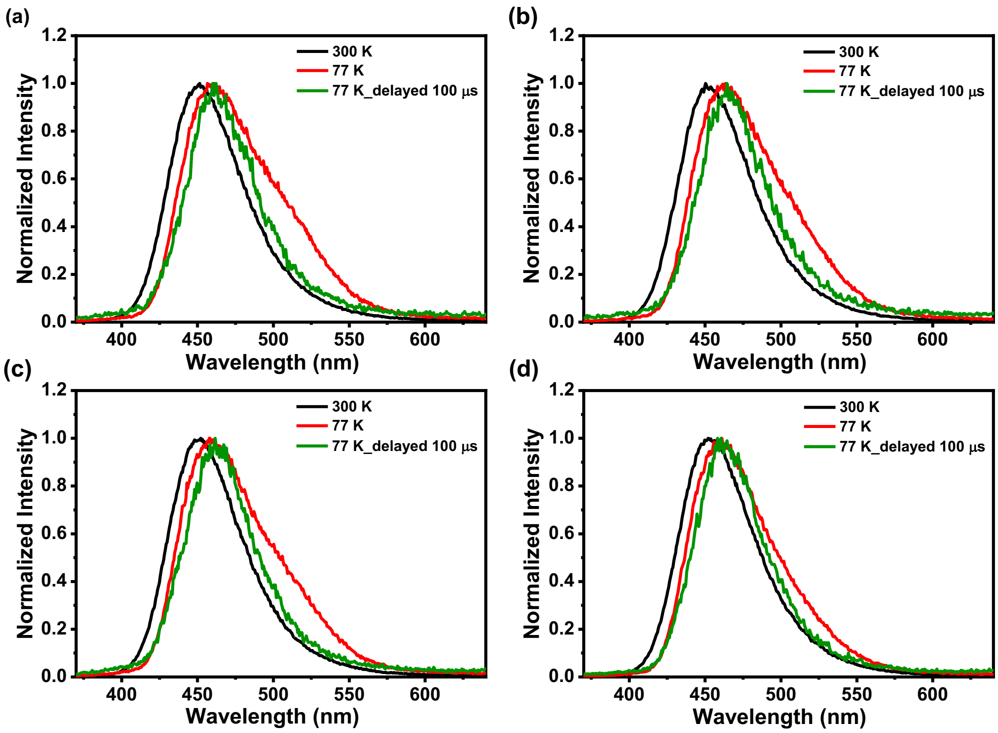

Supplementary Fig. 38 Normalized prompt and delayed emission spectra of 10 wt% (a) **DPA-BO**, (b) **d-DPA-BO**, (c) **DPA-d-BO** and (d) **d-DPA-d-BO** doped in DPEPO films at 300 K and 77 K.

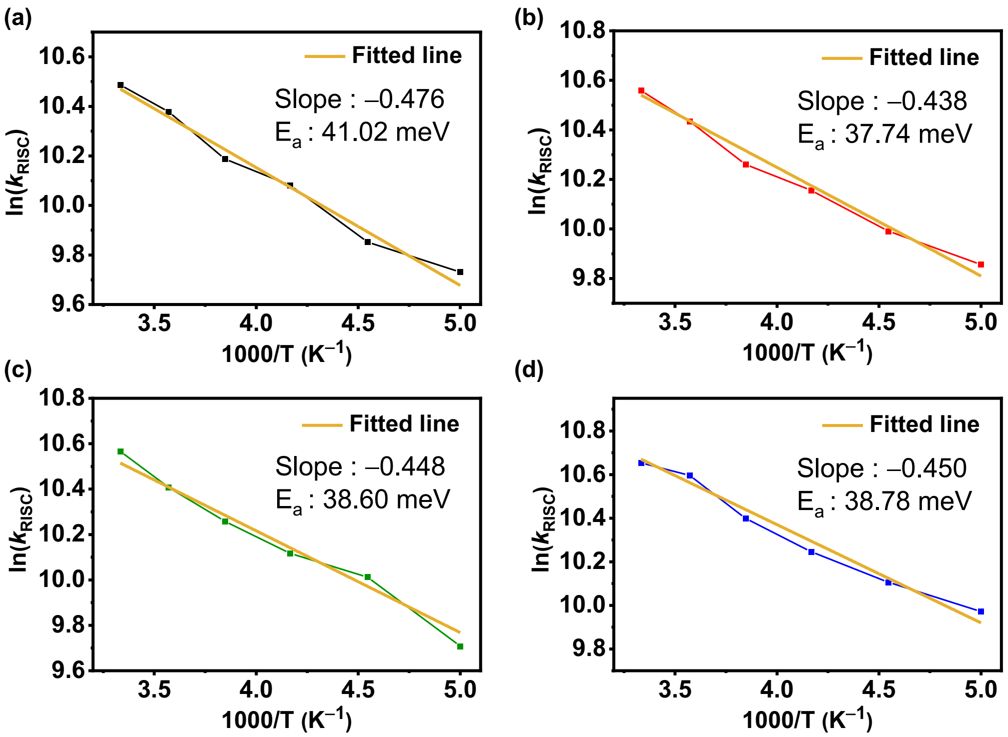

Supplementary Fig. 39 Arrhenius plots of rate constants of RISC (k_{RISC}) of (a) **DPA-BO**, (b) **d-DPA-BO**, (c) **DPA-d-BO** and (d) **d-DPA-d-BO**.

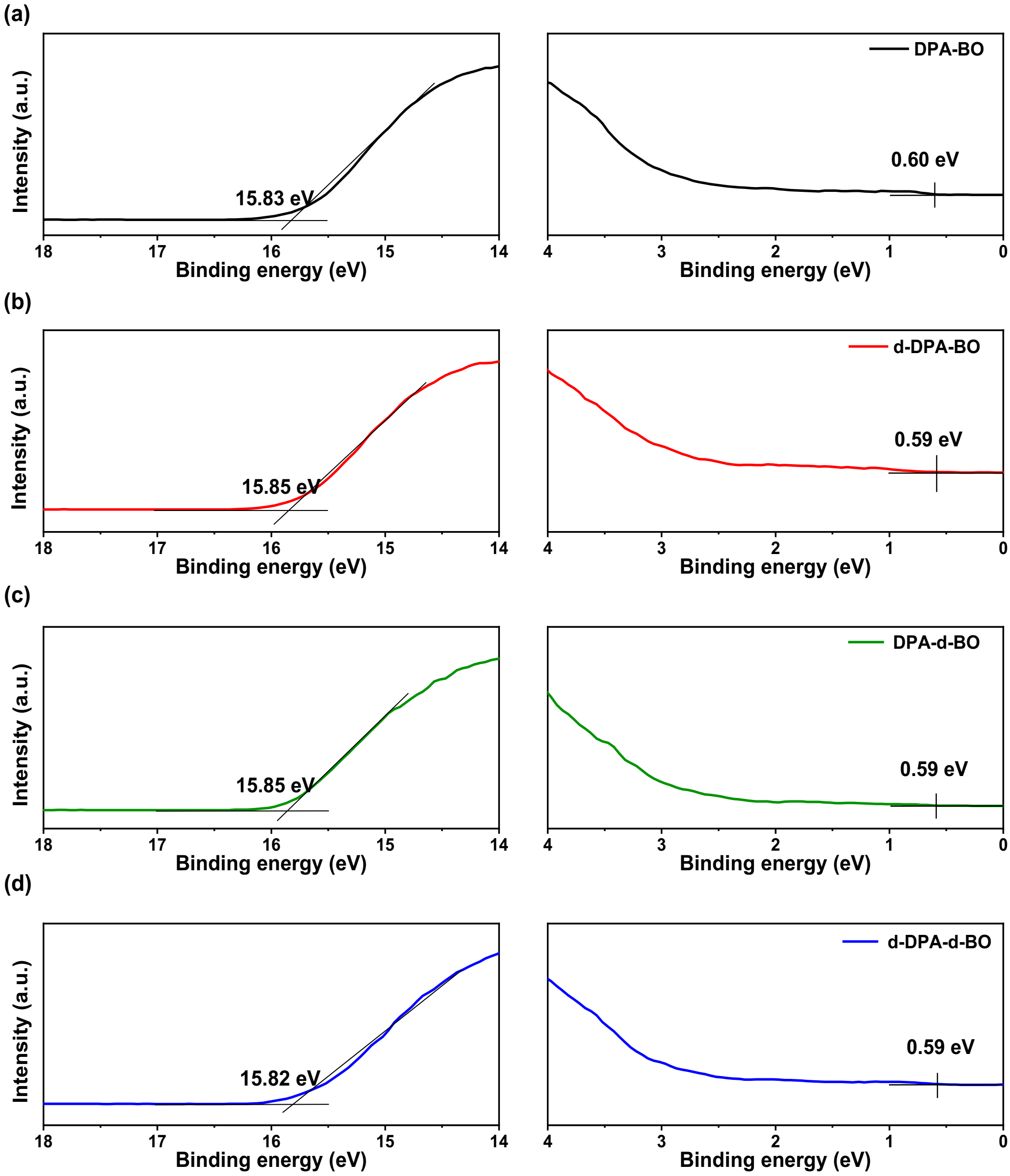

Supplementary Fig. 40 UPS spectra of (a) **DPA-BO**, (b) **d-DPA-BO**, (c) **DPA-d-BO** and (d) **d-DPA-d-BO**.

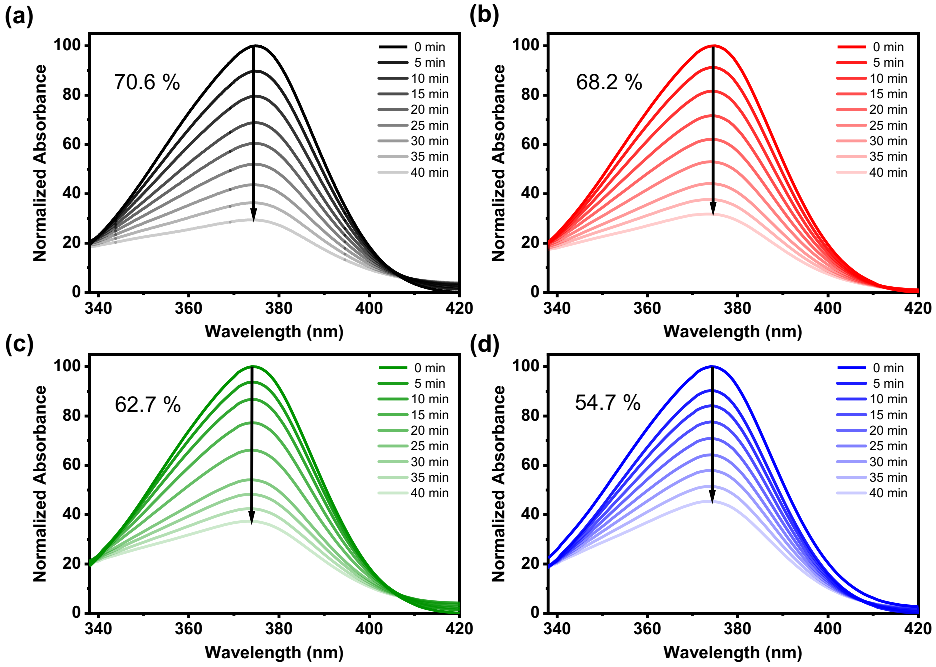

Supplementary Fig. 41 UV-Vis spectra of (a) **DPA-BO**, (b) **d-DPA-BO**, (c) **DPA-d-BO** and (d) **d-DPA-d-BO** at $2{\times}10^{-5}$ M in toluene solution over 40 min under irradiation of 300 W xenon lamp.

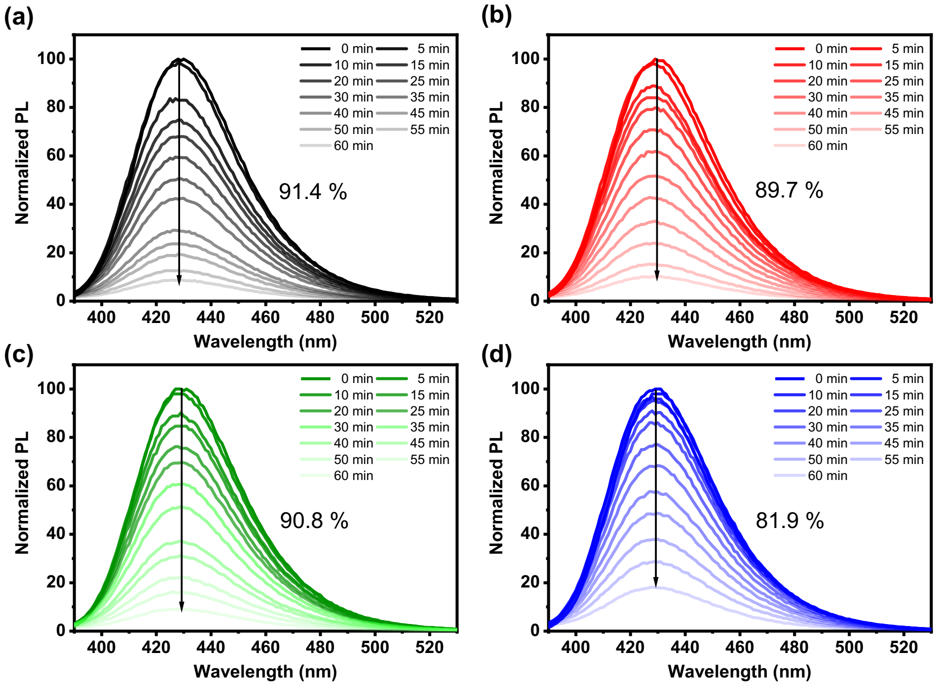

Supplementary Fig. 42 PL spectra of (a) **DPA-BO**, (b) **d-DPA-BO**, (c) **DPA-d-BO** and (d) **d-DPA-d-BO** at $2{\times}10^{-5}$ M in toluene solution over 60 min under irradiation of 300 W xenon lamp.

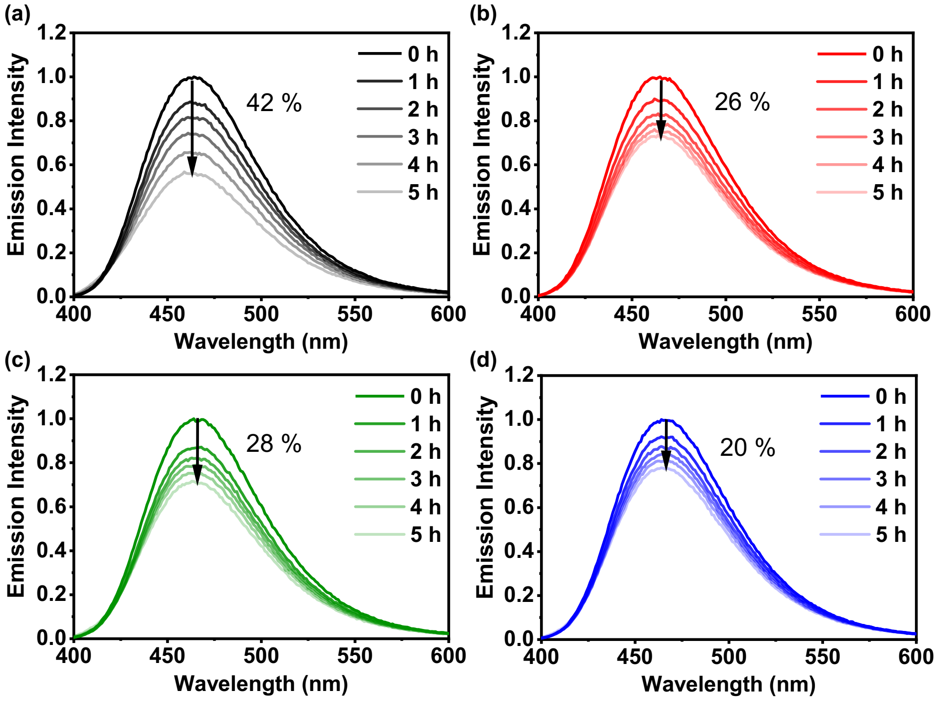

Supplementary Fig. 43 PL spectra of (a) **DPA-BO**, (b) **d-DPA-BO**, (c) **DPA-d-BO** and (d) **d-DPA-d-BO** doped in DPEPO films over 5 h under irradiation of 150 W xenon lamp.

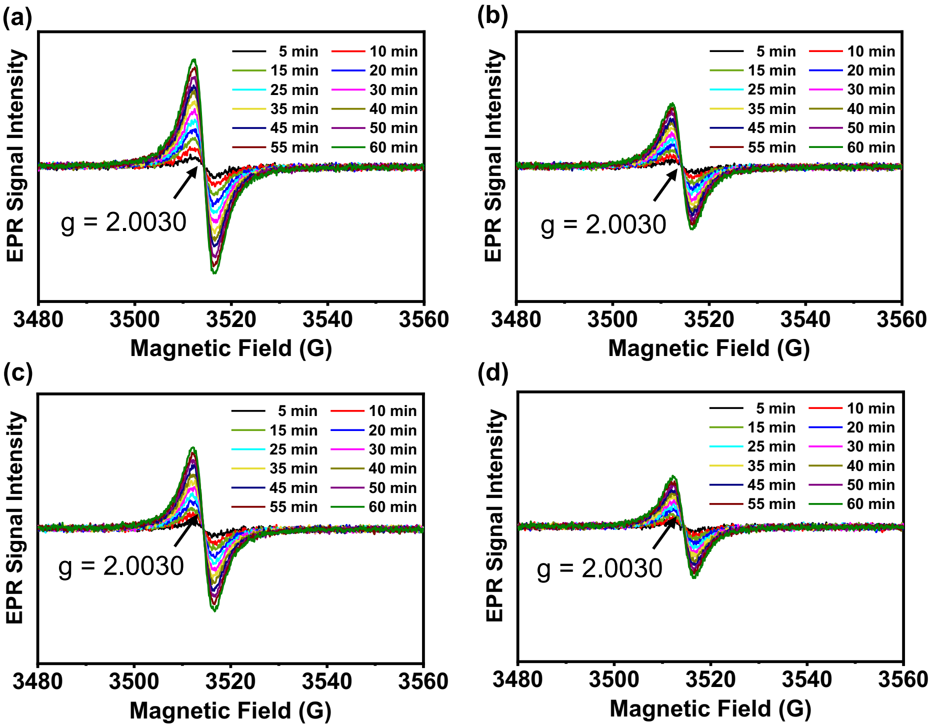

Supplementary Fig. 44 Time-dependent EPR spectra of (a) **DPA-BO**, (b) **d-DPA-BO**, (c) **DPA-d-BO** and (d) **d-DPA-d-BO**.

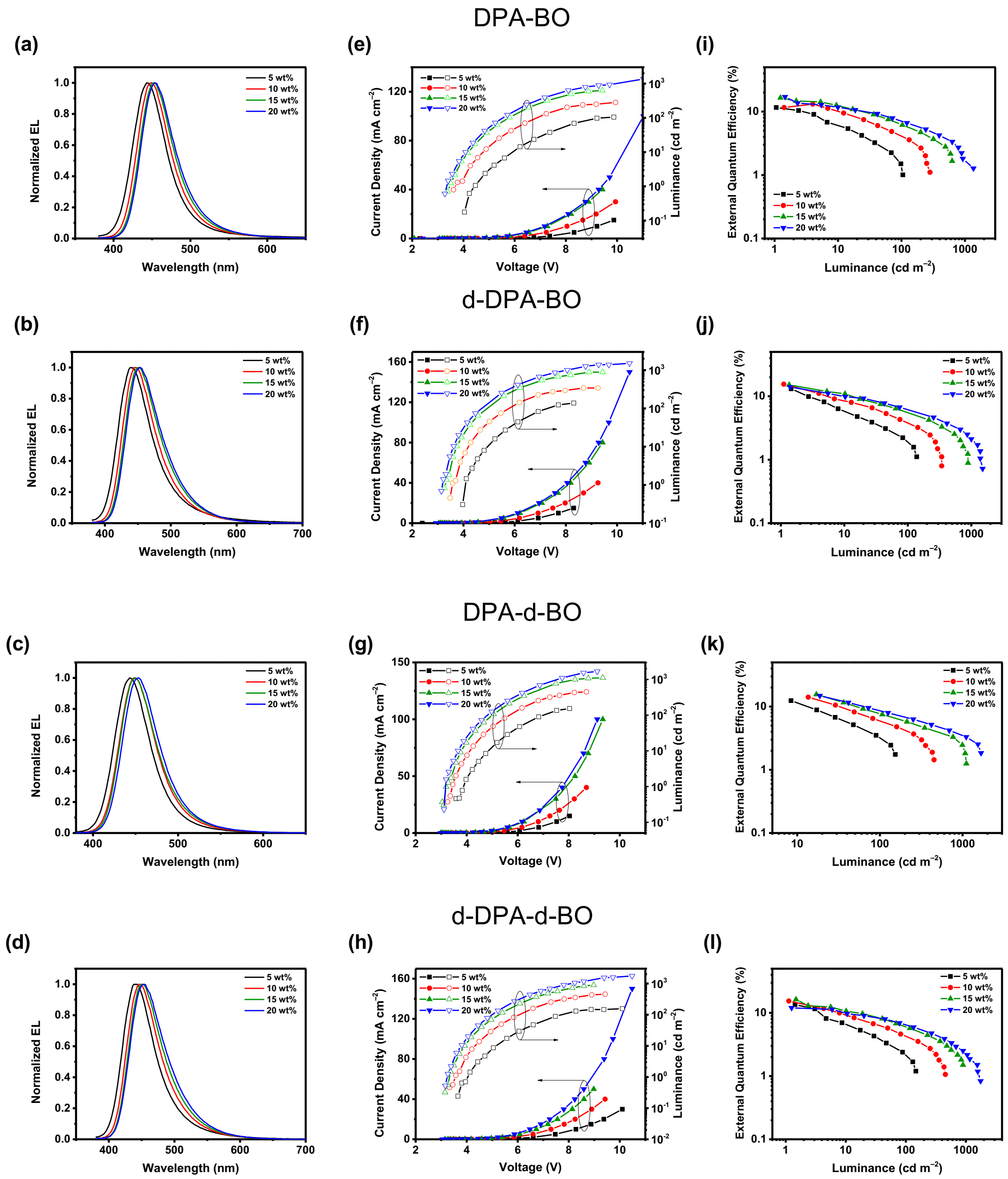

Supplementary Fig. 45 Device characteristics of 5, 10, 15 and 20 wt% of the compounds doped in DPEPO. (a−d) Normalized EL spectra. (e−h) *J–V–L* plots. (i−l) EQE versus luminance.

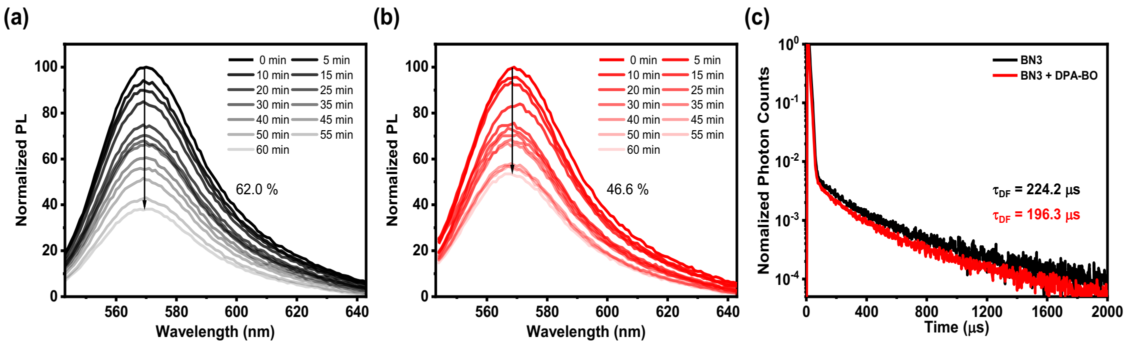

Supplementary Fig. 46 PL spectra of (a) **BN3** (10%) and (b) **BN3** (10%) + **DPA-BO** (15%) doped in mCBP over 60 min under irradiation of 300 W xenon lamp. (c) Delayed PL decay of **BN3** (10%) and **BN3** (10%) + **DPA-BO** (15%) doped in mCBP.

Supplementary Note 1

To allow the conversion of nonradiative triplet excitons to radiative singlet excitons,[7] we attempted to fabricate triplet-triplet upconversion (TTU) organic light-emitting diodes (OLEDs) to study correlation of the intrinsic and operational stability with the compounds doped in 9-(1-naphthalenyl)-10-(4-(2-naphthalenyl)phenyl)anthracene (BH) thin film. Through obtaining the 3D images generated from time-of-flight secondary ion mass spectrometry (ToF-SIMS), we observe the spatial localization of 3 wt% **DPA-BO** doped in BH with a reduced molecular interaction as visualized in **Supplementary Fig. 47**. The device architecture of the TTU OLEDs is shown in **Supplementary Fig. 48a**. The device characteristics are illustrated in **Supplementary Fig. 48b** and **49**, and listed in **Supplementary Table 12**, while the Commission Internationale de l'Éclairage (CIE) chromaticity diagram is shown in **Supplementary Fig. 48c**. Those of other devices are listed in **Supplementary Table 13**. At the optimized 3 wt% dopant concentration, the TTU devices based on these compounds exhibit blue emission peaking at 450 nm, corresponding to the CIE coordinates of (0.15, 0.06), with maximum brightness level of over 10,000 cd m^{-2}. To validate the TTU mechanism, we conduct voltage-dependent electroluminescence (EL) studies and fit the decay curve by the TTU model[7] as shown in **Supplementary Table 14**. The EL lifetimes of all compounds are voltage-dependent and found to decrease from *ca.* 4.3 μs to 1.8 μs upon increasing the driving voltage from 4 V to 8 V (**Supplementary Fig. 48d** and **50**), and the ratio of delayed component is estimated to be *ca.* 30% for all compounds. Considering the radiative exciton ratio of 55% [30% (delayed component) + 25% (singlet)], the photoluminescence quantum yield (PLQY) of *ca.* 60%, the exciton formation efficiency of 100%, and assuming the outcoupling efficiency to be 20−22%, the theoretical external quantum efficiency (EQE) is calculated to be *ca.* 7.0%, in excellent agreement with the experimental EQE of 6.9% at 8 V. In this case, i.e. S_1, T_2 < $2T_1$ < Q_1, the radiative exciton ratio of over 40% might be attributed to the harvesting of triplet excitons from efficient reverse intersystem crossing (RISC) that is mediated by the higher-lying T_n and S_n states.[7,17] Taking advantage of the relatively small EL lifetime of less than 4.3 μs, the devices also show a lower efficiency roll-off of less than 8% at the luminance level of 100 cd m^{-2}.

With the set of TTU OLEDs, we continue to investigate the structure-property relationship between molecular intrinsic stability, based on isotopic effect, and device operational stability, through measuring the operational lifetimes of the encapsulated devices by accelerated testing at a constant driving current of 9 mA under nitrogen. Notably, the device operational stability is found to follow the trend upon increasing the degree of deuteration of the thermally activated delayed fluorescent (TADF) compounds (**Supplementary Fig. 48e**), in which the LT_{50} of the devices are found to be *ca.* 102 h (**DPA-BO**), *ca.* 150−160 h (**d-DPA-BO** and **DPA-d-BO**), and *ca.* 208 h (**d-DPA-d-BO**) at the luminance of 1000 cd m^{-2}, and over 10,000 h at the luminance of 100 cd m^{-2}. It is worth mentioning that we achieve a significant increase of the LT_{90} from 3.1 h (**DPA-BO**) to 10.6 h (**d-DPA-d-BO**) at the luminance of 1000 cd m^{-2} in blue-emitting OLEDs with CIE coordinates of (0.15, 0.06). It should be noted that a shoulder at *ca.* 434 nm could be found in the structureless emission band for 3 wt% **DPA-BO** doped in BH (**Supplementary Fig. 49a**), suggesting possible incomplete energy transfer from BH to the TADF compounds. This is due to the unfortunate fact that it is quite challenging to search for a suitable host material for saturated-blue TADF emitters. Yet, we believe that these results evidence the importance of structural modification at the subatomic level to further boost the device stability regardless of the role of the TADF compounds. To the best of our knowledge, these results represent the longest device lifetimes among blue OLEDs with CIEy ≤ 0.06 (**Supplementary Table 15**). The above results are generally in line with the theoretical and experimental photolysis studies.

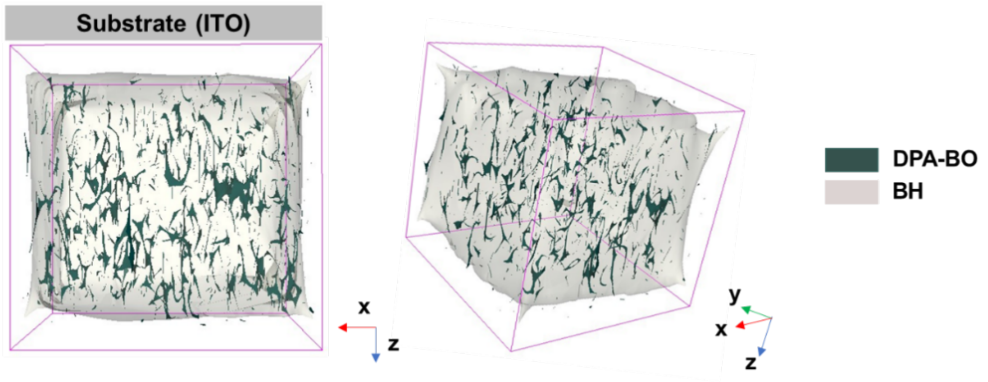

Supplementary Fig. 47 Spatial localization of 3 wt% **DPA-BO** ($C_{30}H_{20}BNO_2^+$, molar mass = 437.16 g/mol) doped in BH ($C_{40}H_{26}^+$, molar mass = 506.20 g/mol).

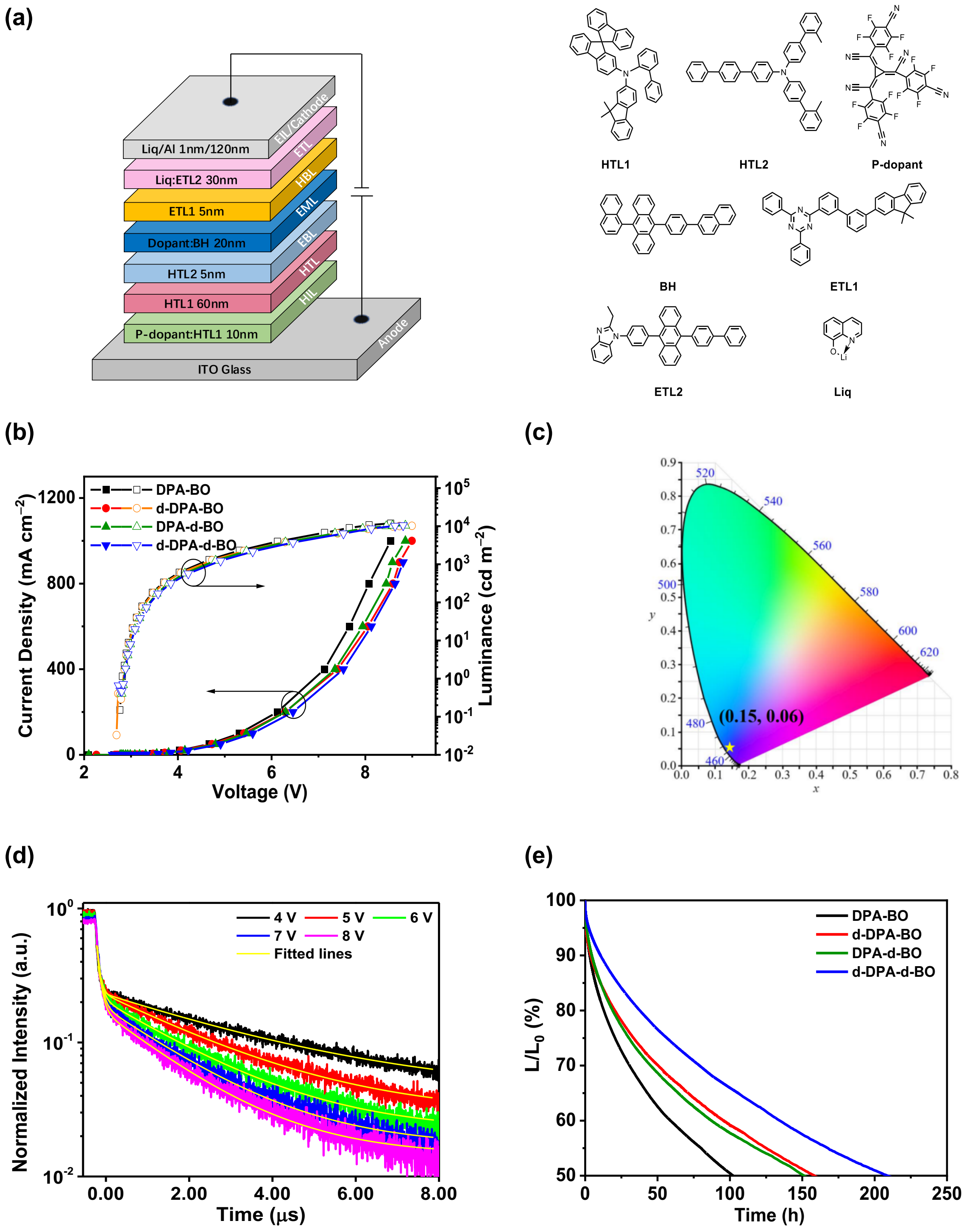

Supplementary Fig. 48 Device characteristics and transient EL decay of vacuum-deposited OLEDs based on the compounds doped in BH. (a) Device configuration and molecular structure of materials used. (b) $J-V-L$ plot of 3 wt% TADF emitters in BH host. (c) CIE chromaticity diagram. (d) Transient EL decay curve of 3 wt% **DPA-BO** fitted with TTU model (yellow lines). (e) Device operational lifetime of all compounds at 3 wt% at projected luminance of 1000 cd m^{-2}.

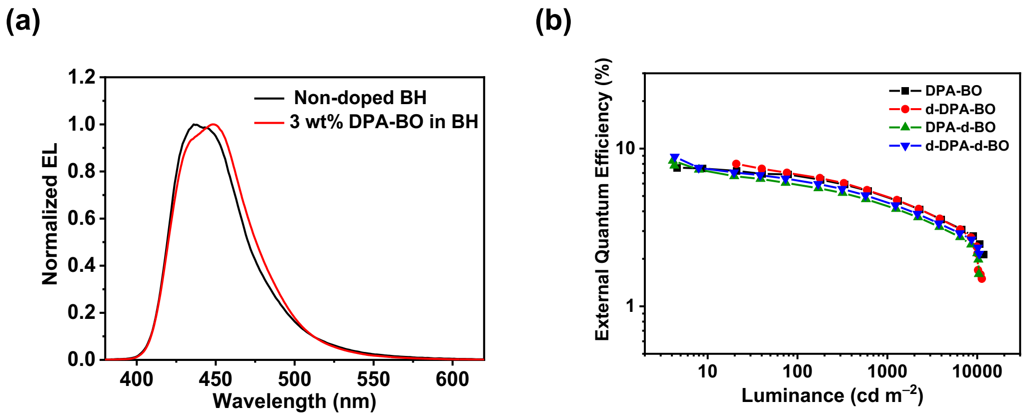

Supplementary Fig. 49 (a) Normalized EL spectra of non-doped BH and 3 wt% **DPA-BO** doped in BH. (b) EQE versus luminance of 3 wt% of the compounds doped in BH.

Supplementary Table 12 Device characteristics of 3 wt% TADF compounds doped in BH.

Compound	V_{on}[a] (V)	L_{max}[b] (cd m^{-2})	λ_{max}[c] (nm)	CIE[d] (x,y)	$EQE_{max/100/1000}$[e] (%)	$\Delta_{roll\text{-}off}$[f] (%)	CE[g] (cd A^{-1})	PE[h] (lm W^{-1})	LT_{90}[i] (h)	LT_{50}[i] (h)
**DPA-BO**	2.8	11852	450	(0.15, 0.06)	7.6/6.7/4.9	11.8	4.6	5.0	3.1	102.2
**d-DPA-BO**	2.8	11256	450	(0.15, 0.06)	8.0/6.9/4.9	13.7	4.2	4.3	3.8	159.1
**DPA-d-BO**	2.8	10412	450	(0.15, 0.06)	7.8/5.9/4.3	24.3	4.3	4.6	4.6	151.7
**d-DPA-d-BO**	2.8	10517	450	(0.15, 0.06)	8.2/6.3/4.5	23.1	4.3	4.6	10.6	208.6

[a)] V_{on} represents turn-on voltage.
[b)] L_{max} represents maximum luminance.
[c)] λ_{max} represents peak maximum.
[d)] CIE coordinates are taken at luminance of 100 cd m^{-2}.
[e)] EQE_{max} represents maximum external quantum efficiency.
[f)] Efficiency roll-off measured by $\Delta_{roll\text{-}off} = 1 - (EQE_{100}/EQE_{max})$.
[g)] CE represents maximum current efficiency.
[h)] PE represents maximum power efficiency.
[i)] Operational lifetime projected at 1000 cd m^{-2}.

Supplementary Table 13 Device characteristics of 1, 3, 5 and 7 wt% of **DPA-BO** doped in BH.

Conc. (wt%)	V_{on}[a] (V)	L_{max}[b] (cd m^{-2})	λ_{max}[c] (nm)	CIE[d] (*x, y*)	$EQE_{max/100/1000}$[e] (%)	$\Delta_{roll\text{-}off}$[f] (%)	CE[g] (cd A^{-1})	PE[h] (lm W^{-1})
1	2.8	13210	450	(0.15, 0.06)	7.4/6.8/4.9	8.1	4.7	4.9
3	2.8	18209	450	(0.15, 0.06)	7.3/6.9/5.4	5.5	4.7	4.9
5	2.8	17162	450	(0.15, 0.06)	7.0/6.7/5.2	4.3	4.4	4.6
7	2.8	16645	450	(0.15, 0.06)	7.0/6.7/5.2	4.3	4.4	4.8

[a)] V_{on} represents turn-on voltage.

[b)] L_{max} represents maximum luminance.

[c)] λ_{max} represents peak maximum.

[d)] CIE coordinates are taken at luminance of 100 cd m^{-2}.

[e)] EQE_{max} represents maximum external quantum efficiency.

[f)] Efficiency roll-off measured by $\Delta_{roll\text{-}off} = 1 - (EQE_{100}/EQE_{max})$.

[g)] CE represents maximum current efficiency.

[h)] PE represents maximum power efficiency.

Supplementary Table 14 Transient EL data of the compounds. The fitting parameters are derived from TTU fitting model and the ratio of delayed TTU contribution is calculated from the composition of prompt and delayed fluorescence EL lifetime.

3 wt% DPA-BO in BH

$y = 1/(At + B)^2$			
**Voltage (V)**	**A**	**B**	**Delayed TTU Ratio (%)**
4	0.10	3.72	7.22
5	0.08	2.62	14.57
6	0.13	2.24	19.93
7	0.11	1.97	25.77
8	0.11	1.79	31.21

3 wt% d-DPA-BO in BH

$y = 1/(At + B)^2$			
**Voltage (V)**	**A**	**B**	**Delayed TTU Ratio (%)**
4	0.11	4.03	6.16
5	0.16	2.77	13.03
6	0.15	2.38	17.65
7	0.12	2.03	24.27
8	0.19	1.77	31.92

3 wt% DPA-d-BO in BH

$y = 1/(At + B)^2$			
**Voltage (V)**	**A**	**B**	**Delayed TTU Ratio (%)**
4	0.09	4.34	5.31
5	0.09	3.33	9.02
6	0.08	2.45	16.66
7	0.08	2.1	22.68
8	0.09	1.81	30.52

3 wt% d-DPA-d-BO in BH

$y = 1/(At + B)^2$			
**Voltage (V)**	**A**	**B**	**Delayed TTU Ratio (%)**
4	0.09	4.00	6.25
5	0.12	2.75	13.22
6	0.14	2.31	18.74
7	0.17	2.11	22.46
8	0.11	1.82	30.19

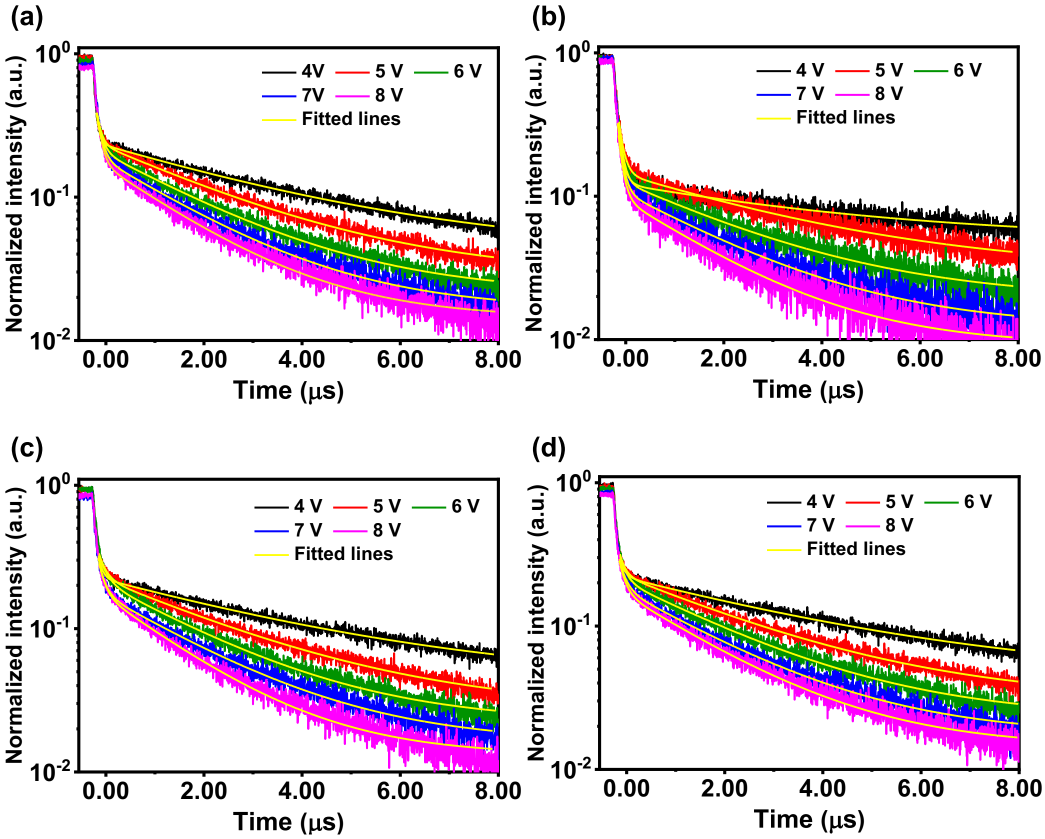

Supplementary Fig. 50 Transient EL decay curves of (a) **DPA-BO,** (b) **d-DPA-BO**, (c) **DPA-d-BO** and (d) **d-DPA-d-BO** fitted with TTU model (yellow lines).

Supplementary Table 15 Key performances of recently reported blue OLEDs with CIE$y \leq 0.1$

Emitter	EQE_{max} (%)	λ_{EL} (nm)	CIE (x, y)	L (cd m^{-2})	LT_x (h)	Ref.
t-Bu-v-DABNA	5.1[c]	462	(0.13, 0.10)	1000	LT_{90} = 19.7	**This work**
t-DABNA	7.6	-	(0.126, 0.098)	200	LT_{90} = 608	1
				1000[f]	LT_{90} = 39	
fac-Ir(pmp)$_3$	10.1	-	(0.16, 0.09)	-	-	2
DABNA-1	13.5	459	(0.13, 0.09)	-	-	3
DABNA-1	13.3	463	(0.13, 0.09)	-	-	4
t-Bu-v-DABNA	5.0[c]	461	(0.13, 0.09)	1000	LT_{90} = 27.3	**This work**
PPCzTrz/v-DABNA[a]	29.3	473	(0.12, 0.09)	1000	LT_{50} = 117	5
PCzTrz/v-DABNA[a]	34.4	473	(0.12, 0.09)	1000	LT_{50} = 97	5
t-DABNA	12.7	-	(x, 0.09)[e]	200	LT_{50} = 17	6
M-tDABNA	6.27	-	(0.137, 0.084)	400	LT_{50} = 70	7
				100[f]	LT_{50} = 739	
PXB-mIC	12.5	-	(0.15, 0.08)	-	-	8
PtON7-dtb	24.8	451	(0.148, 0.079)	-	-	9
DABNA-NP-TB	7.03	458	(0.136, 0.076)	600	LT_{95} = 85	10
2FPPIPCz	5.6	450	(0.150, 0.075)	100[f]	LT_{50} = 49.9	11
DABNA	5.4	-	(0.135, 0.072)	200	LT_{90} = 203	1
				1000[f]	LT_{90} = 13	
DABNA-1	11.3	459	(0.14, 0.07)	-	-	4
TDBA−Ac	21.5	448	(0.15, 0.06)	500	LT_{50} < 2	12
DPA-BO	7.6[d]	450	(0.15, 0.06)	1000	LT_{90} = 3.1	**This work**
					LT_{50} = 102.2	
				100[f]	LT_{50} = 5122	
d-DPA-BO	8.0[d]	450	(0.15, 0.06)	1000	LT_{90} = 3.8	**This work**
					LT_{50} = 159.1	
				100[f]	LT_{50} = 7974	
DPA-d-BO	7.8[d]	450	(0.15, 0.06)	1000	LT_{90} = 4.6	**This work**
					LT_{50} = 151.7	
				100[f]	LT_{50} = 7603	
d-DPA-d-BO	8.2[d]	450	(0.15, 0.06)	1000	LT_{90} = 10.6	**This work**
					LT_{50} = 208.6	
				100[f]	LT_{50} = 10455	
TFPBI[b]	5.74	448	(0.152, 0.054)	100	LT_{50} = 0.55	13
PPI-2BI	4.12	432	(0.154, 0.047)	-	-	14
TPATZ	5.92	430	(0.155, 0.047)	-	-	15
2FPPICz	4.3	425	(0.160, 0.045)	100[f]	LT_{50} = 2.92	11
DMACN-B	10.0	444	(0.151, 0.045)	-	-	16
DFPBI	4.96	425	(0.157, 0.039)	-	-	13

a) Top emitting OLED.

b) Non-doped OLED.

c) TADF-sensitized OLED.

d) TTU OLED.

e) CIEy from ref. 4.

f) Converted using acceleration factor n = 1.7.

Supplementary Table 16 Cartesian coordinates of the optimized S_0 geometry of **DPA-BO**.

1	B	-2.983924	0.000057	0.000043
2	C	-0.730401	-1.173531	0.202678
3	C	-1.472766	0.000064	0.000056
4	C	-0.730417	1.173641	-0.202563
5	C	0.654933	1.198703	-0.211970
6	C	1.356215	0.000114	0.000112
7	C	0.654904	-1.198538	0.212129
8	H	1.177558	2.129886	-0.393071
9	H	1.177714	-2.129572	0.393413
10	C	-2.715075	-2.460782	0.506770
11	C	-3.154149	-3.755965	0.796622
12	C	-3.604865	-1.380089	0.332662
13	C	-4.508602	-3.999246	0.937056
14	H	-2.417337	-4.544203	0.912209
15	C	-4.972812	-1.680257	0.502229
16	C	-5.427388	-2.954127	0.796769
17	H	-4.852825	-5.003957	1.164716
18	H	-6.489676	-3.140956	0.920400
19	C	-2.715047	2.460956	-0.506466
20	C	-3.604820	1.380220	-0.332582
21	C	-3.154087	3.756120	-0.796440
22	C	-4.972752	1.680361	-0.502363
23	C	-4.508519	3.999364	-0.937116
24	H	-2.417260	4.544359	-0.911925
25	C	-5.427306	2.954231	-0.796919
26	H	-4.852743	5.004063	-1.164820
27	H	-6.489578	3.141040	-0.920715
28	H	-5.699726	-0.880627	0.414256
29	H	-5.699664	0.880711	-0.414565
30	O	-1.355598	2.358602	-0.413899
31	O	-1.355640	-2.358451	0.414172
32	N	2.754683	0.000088	-0.000081
33	C	3.481338	1.147392	0.409657
34	C	3.132395	1.829950	1.580043
35	C	4.570569	1.592614	-0.346161
36	C	3.855538	2.949430	1.975683
37	H	2.293540	1.478716	2.172752
38	C	5.299130	2.702451	0.066997
39	H	4.840782	1.062281	-1.254034
40	C	4.943694	3.389635	1.225290
41	H	3.573879	3.471235	2.885707
42	H	6.142882	3.038209	-0.528920
43	H	5.510582	4.259872	1.541586
44	C	3.481057	-1.147393	-0.409884
45	C	4.569912	-1.593185	0.346205
46	C	3.132174	-1.829560	-1.580459
47	C	5.298072	-2.703272	-0.066839
48	H	4.839999	-1.062981	1.254196
49	C	3.854946	-2.949350	-1.976028
50	H	2.293683	-1.477905	-2.173443
51	C	4.942616	-3.390156	-1.225346
52	H	6.141501	-3.039549	0.529241
53	H	3.573285	-3.470900	-2.886191
54	H	5.509241	-4.260595	-1.541565

Supplementary Table 17 Cartesian coordinates of the optimized S_0 geometry of **d-DPA-BO**.

1	B	-2.983912	-0.000091	-0.000019
2	C	-0.730245	-1.173396	0.202271
3	C	-1.472744	0.000072	-0.000410
4	C	-0.730593	1.173765	-0.203231
5	C	0.654712	1.198979	-0.212771
6	C	1.356170	0.000450	-0.000607
7	C	0.655097	-1.198224	0.211634
8	H	1.177375	2.130071	-0.394166
9	H	1.178039	-2.129178	0.392985
10	C	-2.714665	-2.460927	0.506709
11	C	-3.153432	-3.756129	0.796871
12	C	-3.604645	-1.380388	0.332618
13	C	-4.507830	-3.999612	0.937531
14	H	-2.416500	-4.544250	0.912484
15	C	-4.972544	-1.680819	0.502227
16	C	-5.426834	-2.954737	0.797024
17	H	-4.851855	-5.004349	1.165382
18	H	-6.489074	-3.141814	0.920697
19	C	-2.715478	2.460787	-0.506952
20	C	-3.605126	1.380051	-0.332382
21	C	-3.154696	3.755877	-0.796931
22	C	-4.973178	1.680168	-0.501274
23	C	-4.509224	3.999053	-0.936883
24	H	-2.418006	4.544164	-0.912959
25	C	-5.427919	2.953976	-0.795862
26	H	-4.853590	5.003707	-1.164581
27	H	-6.490267	3.140807	-0.918969
28	H	-5.699633	-0.881410	0.413932
29	H	-5.700036	0.880595	-0.412575
30	O	-1.355987	2.358563	-0.414838
31	O	-1.355250	-2.358391	0.413826
32	N	2.754638	0.000557	-0.000813
33	C	3.481262	1.147627	0.409573
34	C	3.131914	1.830038	1.579913
35	C	4.570884	1.592767	-0.345735
36	C	3.855043	2.949368	1.975995
37	H(Iso=2)	2.292803	1.478813	2.172248
38	C	5.299412	2.702446	0.067869
39	H(Iso=2)	4.841300	1.062516	-1.253596
40	C	4.943554	3.389523	1.226105
41	H(Iso=2)	3.573084	3.471083	2.885968
42	H(Iso=2)	6.143455	3.038198	-0.527625
43	H(Iso=2)	5.510438	4.259629	1.542753
44	C	3.481131	-1.147014	-0.410148
45	C	4.569683	-1.592746	0.346318
46	C	3.132583	-1.829358	-1.580779
47	C	5.297946	-2.702937	-0.066387
48	H(Iso=2)	4.839552	-1.062509	1.254353
49	C	3.855389	-2.949194	-1.975981
50	H(Iso=2)	2.294330	-1.477630	-2.174029
51	C	4.942847	-3.389941	-1.224892
52	H(Iso=2)	6.141166	-3.039114	0.530031
53	H(Iso=2)	3.574057	-3.470853	-2.886179
54	H(Iso=2)	5.509508	-4.260439	-1.540861

Supplementary Table 18 Cartesian coordinates of the optimized S_0 geometry of **DPA-d-BO**.

1	B	-2.983906	-0.000032	-0.000030
2	C	-0.730338	-1.173531	0.202353
3	C	-1.472746	0.000025	-0.000252
4	C	-0.730478	1.173678	-0.202873
5	C	0.654841	1.198785	-0.212334
6	C	1.356181	0.000204	-0.000252
7	C	0.654992	-1.198451	0.211831
8	H	1.177581	2.129861	-0.393636
9	H	1.177812	-2.129480	0.393129
10	C	-2.714886	-2.460886	0.506660
11	C	-3.153778	-3.756029	0.796900
12	C	-3.604759	-1.380263	0.332558
13	C	-4.508203	-3.999367	0.937577
14	H(Iso=2)	-2.416920	-4.544211	0.912557
15	C	-4.972683	-1.680545	0.502173
16	C	-5.427100	-2.954403	0.797009
17	H(Iso=2)	-4.852331	-5.004053	1.165497
18	H(Iso=2)	-6.489360	-3.141362	0.920680
19	C	-2.715223	2.460822	-0.506854
20	C	-3.604968	1.380137	-0.332461
21	C	-3.154318	3.755928	-0.796957
22	C	-4.972967	1.680313	-0.501636
23	C	-4.508806	3.999166	-0.937195
24	H(Iso=2)	-2.417561	4.544169	-0.912856
25	C	-5.427581	2.954134	-0.796333
26	H(Iso=2)	-4.853080	5.003827	-1.165007
27	H(Iso=2)	-6.489896	3.141009	-0.919656
28	H(Iso=2)	-5.699700	-0.881055	0.413897
29	H(Iso=2)	-5.699893	0.880766	-0.413118
30	O	-1.355759	2.358540	-0.414412
31	O	-1.355454	-2.358499	0.413754
32	N	2.754640	0.000299	-0.000387
33	C	3.481232	1.147510	0.409703
34	C	3.131907	1.830119	1.579941
35	C	4.570819	1.592584	-0.345694
36	C	3.855004	2.949556	1.975818
37	H	2.292833	1.478971	2.172397
38	C	5.299321	2.702360	0.067697
39	H	4.841284	1.062189	-1.253463
40	C	4.943470	3.389641	1.225817
41	H	3.573038	3.471420	2.885718
42	H	6.143347	3.038035	-0.527889
43	H	5.510334	4.259836	1.542292
44	C	3.481141	-1.147171	-0.409999
45	C	4.569867	-1.592904	0.346228
46	C	3.132391	-1.829439	-1.580604
47	C	5.298111	-2.703027	-0.066722
48	H	4.839917	-1.062749	1.254266
49	C	3.855190	-2.949203	-1.976045
50	H	2.293975	-1.477732	-2.173666
51	C	4.942825	-3.389956	-1.225213
52	H	6.141478	-3.039215	0.529507
53	H	3.573690	-3.470812	-2.886233
54	H	5.509475	-4.260412	-1.541355

Supplementary Table 19 Cartesian coordinates of the optimized S_0 geometry of **d-DPA-d-BO**.

1	B	-2.983869	-0.000073	-0.000058
2	C	-0.730269	-1.173464	0.202455
3	C	-1.472710	0.000042	-0.000284
4	C	-0.730499	1.173700	-0.203090
5	C	0.654816	1.198839	-0.212613
6	C	1.356205	0.000296	-0.000435
7	C	0.655069	-1.198348	0.211841
8	H	1.177493	2.129928	-0.394017
9	H	1.177925	-2.129344	0.393219
10	C	-2.714774	-2.460874	0.506827
11	C	-3.153653	-3.756024	0.797062
12	C	-3.604678	-1.380294	0.332622
13	C	-4.508073	-3.999404	0.937673
14	H(Iso=2)	-2.416760	-4.544170	0.912767
15	C	-4.972603	-1.680601	0.502200
16	C	-5.426998	-2.954467	0.797057
17	H(Iso=2)	-4.852181	-5.004098	1.165582
18	H(Iso=2)	-6.489259	-3.141445	0.920689
19	C	-2.715307	2.460775	-0.506988
20	C	-3.604995	1.380061	-0.332500
21	C	-3.154481	3.755862	-0.797061
22	C	-4.973021	1.680170	-0.501593
23	C	-4.508986	3.999045	-0.937198
24	H(Iso=2)	-2.417747	4.544121	-0.913009
25	C	-5.427709	2.953973	-0.796274
26	H(Iso=2)	-4.853318	5.003693	-1.164971
27	H(Iso=2)	-6.490041	3.140794	-0.919533
28	H(Iso=2)	-5.699627	-0.881130	0.413820
29	H(Iso=2)	-5.699895	0.880589	-0.412978
30	O	-1.355833	2.358529	-0.414675
31	O	-1.355348	-2.358425	0.414017
32	N	2.754680	0.000389	-0.000592
33	C	3.481257	1.147520	0.409731
34	C	3.131833	1.830001	1.580015
35	C	4.570857	1.592724	-0.345570
36	C	3.854845	2.949432	1.976036
37	H(Iso=2)	2.292760	1.478746	2.172400
38	C	5.299278	2.702498	0.067974
39	H(Iso=2)	4.841396	1.062443	-1.253384
40	C	4.943339	3.389640	1.226145
41	H(Iso=2)	3.572803	3.471183	2.885971
42	H(Iso=2)	6.143318	3.038263	-0.527533
43	H(Iso=2)	5.510145	4.259822	1.542742
44	C	3.481171	-1.147111	-0.410145
45	C	4.569925	-1.592789	0.346063
46	C	3.132373	-1.829451	-1.580698
47	C	5.298171	-2.702922	-0.066859
48	H(Iso=2)	4.839983	-1.062590	1.254066
49	C	3.855170	-2.949227	-1.976107
50	H(Iso=2)	2.293936	-1.477788	-2.173742
51	C	4.942846	-3.389918	-1.225297
52	H(Iso=2)	6.141566	-3.039062	0.529348
53	H(Iso=2)	3.573640	-3.470892	-2.886249
54	H(Iso=2)	5.509496	-4.260374	-1.541422

Supplementary Table 20 Cartesian coordinates of the optimized S_1 geometry of **DPA-BO**.

1	B	-2.934855	0.000000	0.000001
2	C	-0.674915	-1.091891	0.491382
3	C	-1.415097	0.000000	0.000001
4	C	-0.674914	1.091891	-0.491381
5	C	0.713371	1.120535	-0.495791
6	C	1.390571	0.000000	0.000002
7	C	0.713371	-1.120535	0.495793
8	H	1.247783	1.982349	-0.880336
9	H	1.247782	-1.982349	0.880339
10	C	-2.675251	-2.338033	0.946143
11	C	-3.109281	-3.574672	1.405853
12	C	-3.554337	-1.330376	0.479809
13	C	-4.472522	-3.871520	1.413601
14	H	-2.370797	-4.291585	1.751708
15	C	-4.922163	-1.690259	0.490812
16	C	-5.377906	-2.922285	0.945513
17	H	-4.815024	-4.836239	1.775877
18	H	-6.441933	-3.143414	0.932787
19	C	-2.675250	2.338034	-0.946141
20	C	-3.554336	1.330377	-0.479808
21	C	-3.109280	3.574674	-1.405851
22	C	-4.922162	1.690260	-0.490812
23	C	-4.472520	3.871521	-1.413600
24	H	-2.370795	4.291586	-1.751705
25	C	-5.377905	2.922287	-0.945513
26	H	-4.815022	4.836241	-1.775876
27	H	-6.441932	3.143415	-0.932788
28	H	-5.649069	-0.979441	0.112338
29	H	-5.649069	0.979442	-0.112339
30	O	-1.301423	2.187991	-0.995451
31	O	-1.301424	-2.187990	0.995453
32	N	2.829272	0.000000	0.000001
33	C	3.492639	0.896002	0.827646
34	C	2.864211	1.334411	2.013043
35	C	4.755259	1.419680	0.470179
36	C	3.512143	2.234457	2.837663
37	H	1.889921	0.937481	2.272611
38	C	5.380792	2.329574	1.303036
39	H	5.202207	1.145579	-0.478850
40	C	4.770589	2.735099	2.492066
41	H	3.035116	2.551958	3.758732
42	H	6.340051	2.746048	1.014058
43	H	5.265752	3.452030	3.138611
44	C	3.492636	-0.896003	-0.827646
45	C	4.755250	-1.419692	-0.470178
46	C	2.864209	-1.334400	-2.013047
47	C	5.380779	-2.329588	-1.303037
48	H	5.202197	-1.145599	0.478854
49	C	3.512136	-2.234447	-2.837670
50	H	1.889922	-0.937461	-2.272616
51	C	4.770577	-2.735101	-2.492071
52	H	6.340034	-2.746070	-1.014058
53	H	3.035110	-2.551940	-3.758742
54	H	5.265736	-3.452033	-3.138618

Supplementary Table 21 Cartesian coordinates of the optimized S_1 geometry of **d-DPA-BO**.

1	B	-2.934863	-0.000002	-0.000004
2	C	-0.674928	-1.091804	0.491539
3	C	-1.415120	-0.000002	-0.000004
4	C	-0.674928	1.091800	-0.491548
5	C	0.713355	1.120453	-0.495949
6	C	1.390569	-0.000002	-0.000002
7	C	0.713355	-1.120457	0.495943
8	H	1.247739	1.982225	-0.880628
9	H	1.247739	-1.982229	0.880623
10	C	-2.675248	-2.337963	0.946276
11	C	-3.109300	-3.574577	1.406040
12	C	-3.554324	-1.330392	0.479723
13	C	-4.472519	-3.871515	1.413570
14	H	-2.370814	-4.291389	1.752103
15	C	-4.922118	-1.690391	0.490434
16	C	-5.377871	-2.922408	0.945163
17	H	-4.815023	-4.836209	1.775912
18	H	-6.441883	-3.143594	0.932205
19	C	-2.675247	2.337961	-0.946281
20	C	-3.554324	1.330390	-0.479727
21	C	-3.109298	3.574577	-1.406041
22	C	-4.922116	1.690392	-0.490432
23	C	-4.472517	3.871517	-1.413566
24	H	-2.370812	4.291388	-1.752105
25	C	-5.377869	2.922411	-0.945157
26	H	-4.815020	4.836213	-1.775905
27	H	-6.441880	3.143598	-0.932195
28	H	-5.648991	-0.979699	0.111648
29	H	-5.648989	0.979700	-0.111645
30	O	-1.301443	2.187818	-0.995829
31	O	-1.301444	-2.187823	0.995818
32	N	2.829261	0.000000	0.000001
33	C	3.492623	0.896008	0.827643
34	C	2.864196	1.334404	2.013043
35	C	4.755262	1.419654	0.470215
36	C	3.512121	2.234441	2.837679
37	H(Iso=2)	1.889914	0.937463	2.272628
38	C	5.380792	2.329540	1.303085
39	H(Iso=2)	5.202239	1.145554	-0.478800
40	C	4.770572	2.735083	2.492100
41	H(Iso=2)	3.035071	2.551934	3.758739
42	H(Iso=2)	6.340069	2.745986	1.014127
43	H(Iso=2)	5.265736	3.452012	3.138644
44	C	3.492628	-0.896007	-0.827639
45	C	4.755267	-1.419650	-0.470209
46	C	2.864204	-1.334404	-2.013041
47	C	5.380801	-2.329533	-1.303078
48	H(Iso=2)	5.202242	-1.145549	0.478807
49	C	3.512133	-2.234440	-2.837676
50	H(Iso=2)	1.889921	-0.937467	-2.272627
51	C	4.770585	-2.735078	-2.492094
52	H(Iso=2)	6.340079	-2.745977	-1.014118
53	H(Iso=2)	3.035085	-2.551935	-3.758736
54	H(Iso=2)	5.265752	-3.452006	-3.138637

Supplementary Table 22 Cartesian coordinates of the optimized S_1 geometry of **DPA-d-BO**.

1	B	-2.934626	-0.000002	-0.000001
2	C	-0.674777	-1.092449	0.490254
3	C	-1.414936	-0.000001	-0.000001
4	C	-0.674779	1.092449	-0.490253
5	C	0.713467	1.121039	-0.494693
6	C	1.390684	0.000002	0.000002
7	C	0.713469	-1.121035	0.494697
8	H	1.247986	1.983187	-0.878356
9	H	1.247988	-1.983182	0.878361
10	C	-2.675283	-2.338348	0.945370
11	C	-3.109529	-3.574881	1.405135
12	C	-3.554329	-1.330042	0.480344
13	C	-4.472960	-3.870850	1.414390
14	H(Iso=2)	-2.371082	-4.292370	1.749879
15	C	-4.922415	-1.688921	0.493110
16	C	-5.378339	-2.920830	0.947898
17	H(Iso=2)	-4.815703	-4.835517	1.776573
18	H(Iso=2)	-6.442514	-3.141341	0.936547
19	C	-2.675285	2.338346	-0.945370
20	C	-3.554331	1.330039	-0.480345
21	C	-3.109533	3.574880	-1.405132
22	C	-4.922417	1.688917	-0.493110
23	C	-4.472964	3.870848	-1.414385
24	H(Iso=2)	-2.371086	4.292371	-1.749875
25	C	-5.378342	2.920826	-0.947894
26	H(Iso=2)	-4.815708	4.835516	-1.776566
27	H(Iso=2)	-6.442517	3.141336	-0.936543
28	H(Iso=2)	-5.649279	-0.977217	0.116232
29	H(Iso=2)	-5.649280	0.977211	-0.116233
30	O	-1.301322	2.189169	-0.993005
31	O	-1.301319	-2.189171	0.993001
32	N	2.829312	0.000003	0.000000
33	C	3.492696	0.895125	0.828585
34	C	2.864279	1.332298	2.014461
35	C	4.755461	1.418966	0.471795
36	C	3.512261	2.231405	2.840058
37	H	1.889971	0.935116	2.273572
38	C	5.381032	2.327907	1.305660
39	H	5.202472	1.145759	-0.477455
40	C	4.770770	2.732303	2.495043
41	H	3.035244	2.547986	3.761449
42	H	6.340377	2.744553	1.017210
43	H	5.265968	3.448512	3.142361
44	C	3.492695	-0.895122	-0.828589
45	C	4.755443	-1.418988	-0.471783
46	C	2.864289	-1.332269	-2.014478
47	C	5.381012	-2.327932	-1.305647
48	H	5.202443	-1.145798	0.477477
49	C	3.512270	-2.231379	-2.840076
50	H	1.889992	-0.935068	-2.273601
51	C	4.770763	-2.732304	-2.495044
52	H	6.340345	-2.744598	-1.017185
53	H	3.035263	-2.547940	-3.761479
54	H	5.265960	-3.448515	-3.142362

Supplementary Table 23 Cartesian coordinates of the optimized S_1 geometry of **d-DPA-d-BO**.

1	B	-2.938228	-0.000009	-0.000010
2	C	-0.675439	-1.135253	0.378644
3	C	-1.417496	-0.000011	-0.000012
4	C	-0.675438	1.135233	-0.378665
5	C	0.712816	1.163924	-0.381080
6	C	1.390477	-0.000011	-0.000010
7	C	0.712816	-1.163946	0.381061
8	H	1.246204	2.060101	-0.678635
9	H	1.246203	-2.060121	0.678623
10	C	-2.676408	-2.379485	0.840731
11	C	-3.109274	-3.620331	1.290236
12	C	-3.557698	-1.322671	0.504251
13	C	-4.474112	-3.865982	1.440768
14	H(Iso=2)	-2.367355	-4.378664	1.521400
15	C	-4.928125	-1.621332	0.687699
16	C	-5.382806	-2.854131	1.141275
17	H(Iso=2)	-4.815437	-4.834737	1.793209
18	H(Iso=2)	-6.449194	-3.025394	1.262447
19	C	-2.676409	2.379471	-0.840742
20	C	-3.557698	1.322654	-0.504266
21	C	-3.109275	3.620317	-1.290244
22	C	-4.928125	1.621315	-0.687715
23	C	-4.474113	3.865968	-1.440777
24	H(Iso=2)	-2.367356	4.378651	-1.521405
25	C	-5.382806	2.854115	-1.141289
26	H(Iso=2)	-4.815438	4.834724	-1.793215
27	H(Iso=2)	-6.449194	3.025378	-1.262462
28	H(Iso=2)	-5.661950	-0.850939	0.477446
29	H(Iso=2)	-5.661951	0.850922	-0.477465
30	O	-1.299750	2.281646	-0.757640
31	O	-1.299750	-2.281662	0.757629
32	N	2.829433	-0.000001	0.000000
33	C	3.493672	0.802510	0.917886
34	C	2.867894	1.111053	2.144845
35	C	4.755159	1.362567	0.615337
36	C	3.518372	1.916664	3.060169
37	H(Iso=2)	1.894305	0.688158	2.362508
38	C	5.382738	2.177923	1.539630
39	H(Iso=2)	5.199637	1.192534	-0.358848
40	C	4.775903	2.452142	2.767230
41	H(Iso=2)	3.044000	2.132777	4.011489
42	H(Iso=2)	6.340912	2.623801	1.294286
43	H(Iso=2)	5.272676	3.095442	3.485956
44	C	3.493709	-0.802498	-0.917876
45	C	4.755163	-1.362594	-0.615263
46	C	2.868008	-1.110983	-2.144887
47	C	5.382784	-2.177935	-1.539541
48	H(Iso=2)	5.199579	-1.192606	0.358957
49	C	3.518527	-1.916581	-3.060195
50	H(Iso=2)	1.894445	-0.688057	-2.362605
51	C	4.776026	-2.452099	-2.767190
52	H(Iso=2)	6.340931	-2.623845	-1.294148
53	H(Iso=2)	3.044213	-2.132649	-4.011554
54	H(Iso=2)	5.272831	-3.095388	-3.485904

Supplementary Table 24 Cartesian coordinates of the optimized T_1 geometry of **DPA-BO**.

1	B	-2.945342	-0.000005	-0.000008
2	C	-0.685699	-1.181574	0.254013
3	C	-1.435873	-0.000007	-0.000011
4	C	-0.685703	1.181556	-0.254034
5	C	0.686484	1.211016	-0.253334
6	C	1.398111	-0.000012	0.000003
7	C	0.686486	-1.211035	0.253327
8	H	1.213851	2.128584	-0.487885
9	H	1.213851	-2.128607	0.487870
10	C	-2.693439	-2.438380	0.648488
11	C	-3.136501	-3.702301	1.022930
12	C	-3.570140	-1.351375	0.430121
13	C	-4.497278	-3.926885	1.214980
14	H	-2.405174	-4.491957	1.165036
15	C	-4.934777	-1.626080	0.658649
16	C	-5.399038	-2.879664	1.039843
17	H	-4.844613	-4.913166	1.508877
18	H	-6.461247	-3.039768	1.201897
19	C	-2.693434	2.438370	-0.648504
20	C	-3.570137	1.351367	-0.430130
21	C	-3.136496	3.702295	-1.022937
22	C	-4.934776	1.626079	-0.658644
23	C	-4.497272	3.926884	-1.214973
24	H	-2.405167	4.491948	-1.165049
25	C	-5.399035	2.879664	-1.039830
26	H	-4.844608	4.913167	-1.508865
27	H	-6.461245	3.039773	-1.201873
28	H	-5.653439	-0.820850	0.549067
29	H	-5.653439	0.820851	-0.549057
30	O	-1.324912	2.357134	-0.509111
31	O	-1.324917	-2.357151	0.509075
32	N	2.793276	-0.000002	0.000000
33	C	3.492249	1.083093	0.558032
34	C	2.976044	1.731853	1.693492
35	C	4.680401	1.544570	-0.036216
36	C	3.651747	2.818077	2.228101
37	H	2.067905	1.356521	2.152556
38	C	5.340788	2.632897	0.509947
39	H	5.054984	1.062263	-0.932851
40	C	4.832922	3.274252	1.642356
41	H	3.258347	3.306907	3.113744
42	H	6.250601	2.994944	0.041508
43	H	5.354706	4.127922	2.063084
44	C	3.492269	-1.083090	-0.558023
45	C	4.680425	-1.544543	0.036231
46	C	2.976082	-1.731860	-1.693484
47	C	5.340838	-2.632858	-0.509927
48	H	5.054994	-1.062229	0.932869
49	C	3.651810	-2.818071	-2.228090
50	H	2.067934	-1.356552	-2.152550
51	C	4.832991	-3.274222	-1.642339
52	H	6.250656	-2.994887	-0.041483
53	H	3.258423	-3.306909	-3.113734
54	H	5.354794	-4.127882	-2.063065

Supplementary Table 25 Cartesian coordinates of the optimized T_2 geometry of **DPA-BO**.

1	B	-3.001967	0.000004	0.000003
2	C	-0.732845	-1.131411	0.320389
3	C	-1.486772	0.000004	0.000001
4	C	-0.732844	1.131421	-0.320391
5	C	0.669881	1.154686	-0.345538
6	C	1.390076	0.000008	-0.000003
7	C	0.669880	-1.154674	0.345531
8	H	1.183014	2.067886	-0.626807
9	H	1.183016	-2.067872	0.626801
10	C	-2.708237	-2.431527	0.678375
11	C	-3.114739	-3.715763	1.032477
12	C	-3.608877	-1.365548	0.371139
13	C	-4.471198	-4.010387	1.097756
14	H	-2.355830	-4.460730	1.249478
15	C	-4.972664	-1.725994	0.449851
16	C	-5.395851	-3.001908	0.801038
17	H	-4.803388	-5.005768	1.372651
18	H	-6.459913	-3.218182	0.845452
19	C	-2.708241	2.431533	-0.678373
20	C	-3.608879	1.365555	-0.371132
21	C	-3.114746	3.715770	-1.032474
22	C	-4.972666	1.726001	-0.449841
23	C	-4.471205	4.010393	-1.097748
24	H	-2.355838	4.460737	-1.249477
25	C	-5.395856	3.001915	-0.801028
26	H	-4.803396	5.005774	-1.372643
27	H	-6.459919	3.218188	-0.845439
28	H	-5.725794	-0.979029	0.225481
29	H	-5.725796	0.979036	-0.225468
30	O	-1.341056	2.290239	-0.651981
31	O	-1.341052	-2.290231	0.651980
32	N	2.795844	0.000003	-0.000001
33	C	3.497204	1.111363	0.521043
34	C	3.041083	1.760745	1.675200
35	C	4.650870	1.584369	-0.116940
36	C	3.723737	2.865049	2.172317
37	H	2.152849	1.393346	2.179904
38	C	5.336138	2.677542	0.399593
39	H	5.004327	1.089523	-1.016025
40	C	4.876591	3.328491	1.542710
41	H	3.356867	3.356778	3.068735
42	H	6.229546	3.031706	-0.106795
43	H	5.410645	4.187051	1.937783
44	C	3.497188	-1.111363	-0.521048
45	C	4.650865	-1.584369	0.116915
46	C	3.041031	-1.760760	-1.675183
47	C	5.336112	-2.677555	-0.399620
48	H	5.004348	-1.089515	1.015985
49	C	3.723664	-2.865077	-2.172300
50	H	2.152785	-1.393363	-2.179868
51	C	4.876532	-3.328517	-1.542717
52	H	6.229530	-3.031718	0.106751
53	H	3.356766	-3.356818	-3.068700
54	H	5.410569	-4.187086	-1.937791

Supplementary Table 26 Cartesian coordinates of the optimized geometry of the dimer of **DPA-BO** at the M06 level.

1	B	-2.974248	0.554762	-0.322829	55	B	-2.011193	0.918488	3.118541
2	C	-0.916441	-0.888000	0.080943	56	C	-4.275334	2.040688	2.796197
3	C	-1.479330	0.352500	-0.248440	57	C	-3.524123	0.907874	3.137929
4	C	-0.577633	1.399285	-0.484101	58	C	-4.252813	-0.245707	3.460555
5	C	0.797150	1.234935	-0.437743	59	C	-5.636895	-0.289542	3.454743
6	C	1.316298	-0.028640	-0.115205	60	C	-6.345893	0.867708	3.105962
7	C	0.449173	-1.099050	0.151932	61	C	-5.660991	2.044337	2.771469
8	H	1.443997	2.081977	-0.644344	62	H	-6.148031	-1.221019	3.678310
9	H	0.823140	-2.072818	0.451590	63	H	-6.191656	2.952329	2.499161
10	C	-3.071646	-1.874649	0.353969	64	C	-2.303384	3.334355	2.442748
11	C	-3.706791	-3.053257	0.750514	65	C	-1.875077	4.607031	2.060213
12	C	-3.788592	-0.729388	-0.040924	66	C	-1.405708	2.294465	2.749625
13	C	-5.087760	-3.120609	0.734750	67	C	-0.522590	4.865526	1.953139
14	H	-3.091529	-3.891088	1.068434	68	H	-2.627602	5.356091	1.828249
15	C	-5.187812	-0.866930	-0.082047	69	C	-0.039665	2.620137	2.650578
16	C	-5.838809	-2.025988	0.298211	70	C	0.406930	3.866509	2.253551
17	H	-5.584938	-4.034731	1.050646	71	H	-0.185641	5.850365	1.637535
18	H	-6.925043	-2.080783	0.266260	72	H	1.473132	4.066235	2.169047
19	C	-2.360718	2.943939	-0.845699	73	C	-2.253845	-1.519919	3.730268
20	C	-3.393296	2.012947	-0.624504	74	C	-1.376465	-0.457861	3.435156
21	C	-2.614306	4.286827	-1.133873	75	C	-1.803998	-2.816080	3.989270
22	C	-4.703162	2.530531	-0.660888	76	C	-0.008516	-0.790489	3.398865
23	C	-3.918732	4.741605	-1.167070	77	C	-0.449689	-3.087084	3.944046
24	H	-1.767636	4.948734	-1.297825	78	H	-2.540527	-3.585504	4.206220
25	C	-4.975056	3.861379	-0.915740	79	C	0.458410	-2.068726	3.644850
26	H	-4.117713	5.789884	-1.378536	80	H	-0.095804	-4.097303	4.136395
27	H	-6.002300	4.218830	-0.913868	81	H	1.523450	-2.281652	3.588757
28	H	-5.777838	-0.027676	-0.441616	82	H	0.694559	1.859522	2.904390
29	H	-5.537389	1.868057	-0.444514	83	H	0.714021	-0.020605	3.138122
30	O	-1.028096	2.643479	-0.782358	84	O	-3.614876	-1.400051	3.777836
31	O	-1.706253	-1.948051	0.387911	85	O	-3.663824	3.207758	2.476534
32	N	2.701227	-0.226970	-0.048032	86	N	-7.750391	0.852157	3.076796
33	C	3.598264	0.852700	0.170803	87	C	-8.505949	0.048012	3.964313
34	C	3.420659	1.725725	1.245885	88	C	-8.147855	-0.064059	5.310941
35	C	4.695076	1.021118	-0.676128	89	C	-9.640345	-0.623017	3.499795
36	C	4.322064	2.761107	1.458658	90	C	-8.906698	-0.848091	6.169380
37	H	2.566983	1.584562	1.906225	91	H	-7.271538	0.466379	5.675660
38	C	5.600164	2.049716	-0.449550	92	C	-10.403279	-1.389891	4.370482
39	H	4.829396	0.334245	-1.509000	93	H	-9.916559	-0.535412	2.451231
40	C	5.416361	2.926721	0.614775	94	C	-10.039448	-1.511773	5.707578
41	H	4.177346	3.431883	2.302963	95	H	-8.616301	-0.927675	7.214392
42	H	6.450629	2.171476	-1.116084	96	H	-11.283861	-1.906161	3.995196
43	H	6.123924	3.733316	0.788632	97	H	-10.634619	-2.118028	6.385536
44	C	3.269438	-1.516990	-0.232477	98	C	-8.440814	1.661981	2.136721
45	C	4.217671	-1.988358	0.677199	99	C	-9.440246	2.540790	2.555015
46	C	2.915711	-2.304996	-1.329669	100	C	-8.125074	1.573122	0.780467
47	C	4.804716	-3.233101	0.489617	101	C	-10.105513	3.327365	1.622267
48	H	4.491125	-1.365106	1.526230	102	H	-9.685845	2.598641	3.613266
49	C	3.494809	-3.555666	-1.501111	103	C	-8.778336	2.375746	-0.145250
50	H	2.181493	-1.929598	-2.038739	104	H	-7.362132	0.860403	0.473902
51	C	4.443072	-4.023973	-0.596292	105	C	-9.772556	3.256329	0.272675
52	H	5.543124	-3.590532	1.203446	106	H	-10.881930	4.011871	1.955613
53	H	3.211678	-4.163074	-2.357460	107	H	-8.519526	2.299044	-1.199477
54	H	4.899224	-5.000352	-0.738333	108	H	-10.290258	3.880227	-0.451426

Supplementary References

1. Lee, K. H. & Lee, J. Y. Paradigm change of blue emitters: Thermally activated fluorescence emitters as long-living fluorescence emitters by triplet exciton quenching. *Org. Electron.* **75**, 105377 (2019).
2. Lee, J. *et al.* Deep blue phosphorescent organic light-emitting diodes with very high brightness and efficiency. *Nat. Mater.* **15**, 92−98 (2016).
3. Hatakeyama, T. *et al.* Ultrapure blue thermally activated delayed fluorescence molecules: efficient HOMO-LUMO separation by the multiple resonance effect. *Adv. Mater.* **28**, 2777−2781 (2016).
4. Wu, X. *et al.* The role of host–guest interactions in organic emitters employing MR-TADF. *Nat. Photon.* **15**, 780−786 (2021).
5. Jeon, S. O. *et al.* High-efficiency, long-lifetime deep-blue organic light-emitting diodes. *Nat. Photon.* **15**, 208−215 (2021).
6. Lee, K. H. & Lee, J. Y. Phosphor sensitized thermally activated delayed fluorescence organic light-emitting diodes with ideal deep blue device performances. *J. Mater. Chem. C* **7**, 8562−8568 (2019).
7. Lim, H., Woo, S.-J., Ha, Y. H., Kim, Y.-H., Kim, J.-J. Breaking the Efficiency Limit of Deep-Blue Fluorescent OLEDs Based on Anthracene Derivatives. *Adv. Mater.* **34**, 2100161 (2022).
8. Ahn, D. H. *et al.* Highly twisted donor-acceptor boron emitter and high triplet host material for highly efficient blue thermally activated delayed fluorescent device. *ACS Appl. Mater. Interfaces* **11**, 14909−14916 (2019).
9. Fleetham, T., Li, G., Wen, L. & Li, J. Efficient "pure" blue OLEDs employing tetradentate Pt complexes with a narrow spectral bandwidth. *Adv. Mater.* **26**, 7116−7121 (2014).
10. Cho, S. M. *et al.* Anthracene-dibenzofuran based electron transport type hosts for long lifetime multiple resonance pure blue OLEDs. *Org. Electron.* **105**, 106501 (2022).

11. Xin, J. *et al.* High-efficiency non-doped deep-blue fluorescent organic light-emitting diodes based on carbazole/phenanthroimidazole derivatives. *J. Mater. Chem. C* **8**, 10185−10190 (2020).
12. Ahn, D. H. *et al.* Highly efficient blue thermally activated delayed fluorescence emitters based on symmetrical and rigid oxygen-bridged boron acceptors. *Nat. Photon.* **13**, 540−546 (2019).
13. Qiu, X. *et al.* Novel 9,9-dimethylfluorene-bridged D–π–A-type fluorophores with a hybridized local and charge-transfer excited state for deep-blue electroluminescence with $CIE_y \sim 0.05$. *J. Mater. Chem. C* **7**, 592−600 (2019).
14. Liu, B. *et al.* Ternary acceptor-donor-acceptor asymmetrical phenanthroimidazole molecule for highly efficient near-ultraviolet electroluminescence with external quantum efficiency (EQE) >4 %. *Chem. Eur. J.* **24**, 15566−15571 (2018).
15. Shi, J. *et al.* A simple D–π–A hybrid mode for highly efficient non-doped true blue OLEDs with $CIE_y < 0.05$ and EQE up to 6%. *J. Mater. Chem. C* **6**, 11063−11070 (2018).
16. Khan, A. *et al*. Intramolecular-locked high efficiency ultrapure violet-blue (CIE-y <0.046) thermally activated delayed fluorescence emitters exhibiting amplified spontaneous emission. *Adv. Funct. Mater.* **31**, 2009488 (2021).
17. Ieuji, R., Goushi, K. & Adachi, C. Triplet–triplet upconversion enhanced by spin–orbit coupling in organic light-emitting diodes. *Nat. Commun*. **10**, 5283 (2019).
